# Novel HSP90 inhibitors, NVP-AUY922 and NVP-BEP800, radiosensitise tumour cells through cell-cycle impairment, increased DNA damage and repair protraction

**DOI:** 10.1038/sj.bjc.6605683

**Published:** 2010-05-25

**Authors:** L Stingl, T Stühmer, M Chatterjee, M R Jensen, M Flentje, C S Djuzenova

**Affiliations:** 1 Klinik für Strahlentherapie der Universität Würzburg, Josef-Schneider-Strasse 11, D-97080 Würzburg, Germany; 2 Medizinische Klinik und Poliklinik II der Universität Würzburg, Oberdürrbacher Strasse 6, D-97080 Würzburg, Germany; 3 Oncology Research, Novartis Institutes for Biomedical Research, Novartis Pharma AG, CH-4057 Basel, Switzerland

**Keywords:** colony survival, DNA damage, cell-cycle arrest, histone *γ*H2AX

## Abstract

**Background:**

Heat-shock protein 90 (Hsp90) has a crucial role in both the stabilisation and regulation of various proteins, including those related to radioresistance. Inhibition of Hsp90 may therefore provide a strategy for enhancing the radiosensitivity of tumour cells. This study explores the responses of four tumour cell lines (A549, GaMG, HT 1080 and SNB19) to combined treatment with ionising radiation (IR) and two novel inhibitors of Hsp90, NVP-AUY922 and NVP-BEP800. The techniques used included cell and colony counts, expression of Hsp90, Hsp70, Akt, survivin, cleaved caspase 3, p53, cell-cycle progression and associated proteins. DNA damage was analysed by histone *γ*H2AX and Comet assays.

**Results:**

We found that NVP-AUY922 and NVP-BEP800 enhanced radiosensitivity in all tested cell lines. In contrast, only two cell lines (HT 1080 and GaMG) exhibited an increased rate of apoptosis after drug pretreatment, as revealed by western blot. In all tested cell lines, the expression of histone *γ*H2AX, a marker of DNA double-strand breaks, after combined drug-IR treatment was higher and its decay rate was slower than those after each single treatment modality. Drug-IR treatment also resulted in impaired cell-cycle progression, as indicated by S-phase depletion and G2/M arrest. In addition, the cell cycle-associated proteins, Cdk1 and Cdk4, were downregulated after Hsp90 inhibition.

**Interpretation:**

These findings show that the novel inhibitors of Hsp90 can radiosensitise tumour cell lines of different entities through destabilisation and depletion of several Hsp90 client proteins, thus causing the depletion of S phase and G2/M arrest, increased DNA damage and repair protraction and, to some extent, apoptosis. The results might have important implications for the radiotherapy of solid tumours.

Heat-shock proteins 90 (Hsp90s) are ubiquitously and abundantly expressed polypeptides required for the energy-driven stabilisation, conformation and function of a large number of cellular proteins, termed Hsp90 clients (Picard, 2002; Whitesell and Lindquist, 2005). Several key Hsp90 clients are involved in the processes characteristic to the malignant phenotype, such as invasion, angiogenesis and metastasis (Neckers, 2002). Hsp90 clients also contribute to the pathways leading to the induction of mitogen-activated protein kinases (MAPK) and nuclear factor-kappa B (NF-*κ*B) (Sato *et al*, 2003; Mitsiades *et al*, 2006; Chatterjee *et al*, 2007). Moreover, Hsp90 stabilises Raf-1, Akt and ErbB2 proteins (Schulte *et al*, 1995; Sato *et al*, 2000; Bull *et al*, 2004), which are known to be associated with protection against radiation-induced cell death (Pirollo *et al*, 1997; Gupta *et al*, 2001; Tanno *et al*, 2004).

The diverse molecular functions of Hsp90 suggest that its inhibitors could provide a promising strategy for implementing a multitarget approach to radiosensitisation. Indeed, a number of studies have already explored Hsp90 as a potential molecular target for radiosensitisation of tumour cells (Bisht *et al*, 2003; Machida *et al*, 2003; Russell *et al*, 2003; Bull *et al*, 2004; Harashima *et al*, 2005; Dote *et al*, 2006; Kabakov *et al*, 2008; Wu *et al*, 2009). Thus, the inhibitor of Hsp90, geldanamycin, and its derivatives significantly enhance the radiosensitivity of tumour cell lines derived from a variety of histologies, including glioma, prostate, pancreas and cervix (Bisht *et al*, 2003; Enmon *et al*, 2003; Machida *et al*, 2003; Russell *et al*, 2003; Bull *et al*, 2004; Harashima *et al*, 2005; Dote *et al*, 2006). However, geldanamycins have several limitations, including poor solubility, formulation difficulties, hepatotoxicity and extensive metabolism by polymorphic enzymes, along with drug efflux by P-glycoprotein (Kelland *et al*, 1999; Eiseman *et al*, 2005; Brough *et al*, 2008; Eccles *et al*, 2008). Therefore, there has been considerable effort to design small synthetic inhibitors of Hsp90 with improved bioavailability and lower toxicity. Both requirements are met by a series of pyrazole resorcinol compounds that have proven to be stronger inhibitors of Hsp90 than geldanamycin derivatives. Currently, the isoxazole resorcinol NVP-AUY922 shows the highest affinity for the NH_2_-terminal nucleotide-binding site of Hsp90 (Brough *et al*, 2008; Eccles *et al*, 2008; Gao *et al*, 2010), whereas NVP-BEP800 represents a novel fully synthetic, orally available 2-aminothieno[2,3-*d*]pyrimidine class Hsp90 inhibitor (Brough *et al*, 2009; Stühmer *et al*, 2009; Massey *et al*, 2010). Both compounds have good pharmaceutical and pharmacological properties. They also exhibit strong anti-proliferative activity against various tumour cell lines and primary tumours *in vitro* and *in vivo* at well-tolerated doses (Eccles *et al*, 2008; Jensen *et al*, 2008; Stühmer *et al*, 2009; Massey *et al*, 2010).

This study explores the cytotoxicity and radiosensitising ability of NVP-AUY922 and NVP-BEP800 in four established cell lines originated from different tumour entities, including lung carcinoma A549, fibrosarcoma HT 1080, and two glioblastoma, SNB19 and GaMG, cell lines. Each tumour cell line was treated with drug, ionising radiation (IR) or combined drug-IR exposure. Treated cells were then analysed for proliferation rate, colony-forming ability, cell-cycle distribution and expression of several marker proteins (Hsp90, Hsp70, Akt, phospho-Akt, survivin, p53, cleaved caspase 3, Cdk1, Cdk2, Cdk4, pRb and so on). In addition, radiation-induced DNA damage and repair were assessed by histone *γ*H2AX and Comet assay.

## Materials and methods

### Cells

The group of human tumour cell lines examined includes lung carcinoma A549 (p53wt), fibrosarcoma HT 1080 (p53wt) and two glioblastomas, namely, GaMG (p53mt) and SNB19 (p53mt). Cells were obtained from the American Type Culture Collection (Manassas, VA, USA) and routinely cultured under standard conditions (5% CO_2_, 37°C) in complete growth medium (CGM), which was either MEM (GaMG, SNB19) or DMEM (A549, HT 1080), supplemented with 10% foetal bovine serum.

### Drug treatment

NVP-AUY922 (Brough *et al*, 2008) and NVP-BEP800 (Brough *et al*, 2009) were kindly provided by Novartis Institutes for Biomedical Research (Basel, Switzerland). 17-Dimethylaminoethylamino-17-demethoxygeldanamycin (17-DMAG) was purchased from Sigma (Taufkirchen, Germany, D-5193). Drugs were freshly diluted from frozen aliquots in DMSO stored at −20°C. Exponentially growing cell cultures were incubated with different concentrations of NVP-AUY922, NVP-BEP800 or 17-DMAG, added to CGM for 24 h. Thereafter, CGM was aspirated, and the cell monolayers were rinsed with PBS, which was then replaced by fresh drug-free CGM. Control cells were treated in parallel with respective concentrations of DMSO as a vehicle control.

### Growth inhibition assay

The growth inhibition assay was carried out essentially as described elsewhere (Robles *et al*, 2006). Serial dilutions of Hsp90 inhibitors (0–5 *μ*M) in CGM were added to cell cultures in duplicates. The cytotoxicity of each drug was determined 24 h later (Robles *et al*, 2006) using the Cell Titer 96 Aqueous One Solution Cell Proliferation Assay (Promega, Madison, WI, USA) according to the manufacturer's instructions. Control samples contained the respective concentrations of DMSO. Duplicate data from two independent experiments were averaged and normalised against non-treated controls to generate dose–response curves.

### Antibodies

The primary and secondary antibodies used are specified in Supplementary Information.

### X-ray irradiation

Irradiation was performed at room temperature using a 6 MV Siemens linear accelerator (Siemens, Concord, CA, USA) at a dose rate of 2 Gy min^−1^. After irradiation, cells were recovered in CGM for the indicated time until harvest.

### Colony survival

Cell survival curves were generated by a standard colony formation assay as previously described (Djuzenova *et al*, 2004). Subconfluent monolayers of non-treated and drug-treated cells were irradiated in culture flasks filled with CGM at room temperature by graded single doses (0–8 Gy), seeded in Petri dishes and then cultivated in CGM for the next 2 weeks. Four replications were carried out for each exposure point, and the experiments were repeated at least twice. After 2 weeks, the cells were fixed and stained with crystal violet (0.6%). Colonies of at least 50 cells were scored as survivors. The mean survival data for each individual cell line were fitted to the linear quadratic (LQ) model: 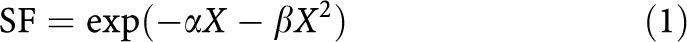 where, SF is the survival fraction, *X* is the irradiation dose and *α* and *β* are the fitted parameters.

### Western blot

For immunoblot analysis, whole-cell lysates were prepared according to standard procedures. Samples equivalent to 10–100 *μ*g of protein were separated using 4–12% or 3–8% SDS–polyacrylamide precast gels (Invitrogen, Karlsruhe, Germany) and transferred to nitrocellulose membranes according to the manufacturer's prescriptions. For protein detection, membranes were incubated with respective primary and species-specific peroxidase-labelled secondary antibodies according to standard protocols. The levels of protein expression were quantified using Kodak 1D Image analysing software (Scientific Imaging Systems, Eastman Kodak Company, Rochester, NY, USA) and normalised to the *β*-actin levels.

### Comet assay

Comet assay was performed under alkaline conditions following the protocol reported elsewhere (Djuzenova *et al*, 2006). Just before irradiation, drug-treated and control cells were embedded in a thin layer of agarose spread on glass microscope slides. The slides were placed on ice, subjected to irradiation and transferred immediately either into ice-cold lysis buffer or to CGM for the indicated times. DNA fragmentation was quantified from the ‘Tail Moment’ (TM, given in arbitrary units, a.u.) defined as the product of the percentage of DNA in the comet tail and the tail length (Olive *et al*, 1990).

### Immunocytochemical detection of histone *γ*H2AX and cell-cycle measurements by flow cytometry

Non-treated and drug-treated cell cultures were irradiated as subconfluent monolayers in CGM at room temperature. The cells were then incubated in the same medium under standard conditions and analysed by flow cytometry 30 min, 1 and 2 days after IR exposure. For analysis, cells were trypsinised, washed twice in PBS, fixed and stained for *γ*H2AX, according to a protocol described elsewhere (Muslimovic *et al*, 2008). The cells were then counterstained with propidium iodide (Sigma P-4170, 10 *μ*g ml^−1^) in the presence of ribonuclease A (Sigma R-5250, 25 *μ*g ml^−1^) as described elsewhere (Djuzenova *et al*, 1994).

At least 15 000 cells were assayed for either histone *γ*H2AX or DNA distribution using a flow cytometer FACSCalibur (Becton Dickinson, San Jose, CA, USA) equipped with a 15 mW argon-ion laser. Cellular green (histone *γ*H2AX) or red fluorescence (staining with propidium iodide) was acquired in logarithmic or linear mode. The output data presented as one-dimensional histograms, that is, the distributions of histone *γ*H2AX or PI-DNA signals within cell samples, were analysed using the WinMDI program obtained from J. Trotter (The Scripps Research Institute, La Jolla, CA, USA) and the ModFit LT program (Verity Software House, Topsham, ME, USA).

### Statistics

Data are presented as means (±s.d. or ±s.e.). Mean values were compared by Student's *t-*test. The threshold of statistical significance was set at *P*<0.05. Statistics and fitting of experimental curves were performed with the program Origin (Microcal, Northampton, MA, USA).

## Results

### Effects of Hsp90 inhibitors on cell growth and radiosensitivity

**Figure 1:**
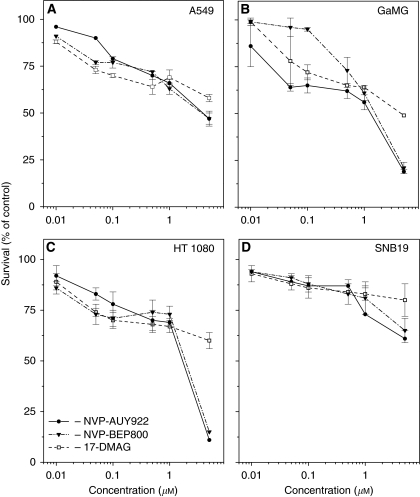
Cytotoxicity assay after exposure of tumour cell lines (**A**, A549; **B**, GaMG; **C**, HT 1080; and **D**, SNB19) to serial dilutions of NVP-AUY922 (filled circles), NVP-BEP800 (triangles) and 17-DMAG (squares), for 24 h. Survival was measured by standard MTT assay. Triplicate data from two experiments were averaged and normalised against non-treated controls (DMSO) to generate dose–response curves. The numbers of viable cells are expressed as percentage (mean±s.d.) relative to corresponding controls.

We first analysed the effects of Hsp90 inhibitors on the growth of tumour cell lines. To this end, we treated cells for 24 h with different drug concentrations ranging from 0 to 5 *μ*M, and then analysed cell viability by MTT assay. As seen in Figure 1, GaMG and HT 1080 cell lines were more sensitive to high concentrations (5 *μ*M) of Hsp90 inhibitors than were A549 and SNB19 cells. Dose–response curves show that, at a concentration of ∼200 nM, all tested drugs yielded ∼70% viability in all cell lines. For that reason, the drugs were used at the same concentration of 200 nM in subsequent experiments. Besides this, the selected drug concentration is consistent with the previously reported 100 nM for 17-DMAG (Robles *et al*, 2006; Koll *et al*, 2008).

**Figure 2:**
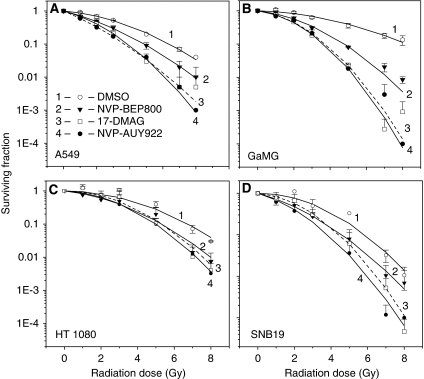
Clonogenic abilities of tumour cell lines (**A**, A549; **B**, GaMG; **C**, HT 1080; and **D**, SNB19) as functions of radiation dose and drug exposure. Control (DMSO treated, empty circles) and drug-treated (NVP-AUY922 – filled circles; NVP-BEP800 – triangles; 17-DMAG – squares) cells were irradiated with single ionising radiation (IR) doses ranging between 1 and 8 Gy. After irradiation, cells were plated in CGM and incubated under standard conditions. After 2 weeks, colonies containing at least 50 cells were scored as survivors. Data derived from at least two independent experiments for each cell line were pooled together and fitted by a linear quadratic equation (equation (1)). The s.d. values are indicated by error bars.

On the basis of the cytotoxicity data shown in Figure 1, drug-pretreated (200 nM, 24 h) cells were exposed to an X-ray dose of up to 8 Gy and their radiation sensitivities were analysed by means of the colony survival test. Figure 2 shows the normalised cell survival responses (symbols) plotted *vs* the X-ray dose, along with the best fits of the LQ model (equation (1)) to the data. Judging by the correlation coefficients, which range between 0.97 and 0.99, the LQ model provides reasonable approximations to the experimental data. The plating efficiencies of non-irradiated cell lines and the fitted parameters *α* and *β* obtained by non-linear regression of the LQ model are summarised in Table 1 for each individual cell line. The table also includes data for the surviving cell fractions at 2 Gy (SF2) and the radiation doses (D_10_) resulting in 10% survival. Comparison of the SF2 and D_10_ values of drug-treated cell samples with the corresponding data of untreated controls (Table 1) reveals a marked drug-induced reduction of both SF2 and D_10_ values in four cell lines. The data shown in Figure 2 and Table 1 prove the three tested Hsp90 inhibitors (NVP-AUY922, NVP-BEP800 and 17-DMAG) as potent radiosensitisers that significantly enhance *in vitro* radiotoxicity, regardless of the p53 status of the particular tumour line.

### Effects of Hsp90 inhibition and/or radiation on multiple signalling pathways

**Figure 3:**
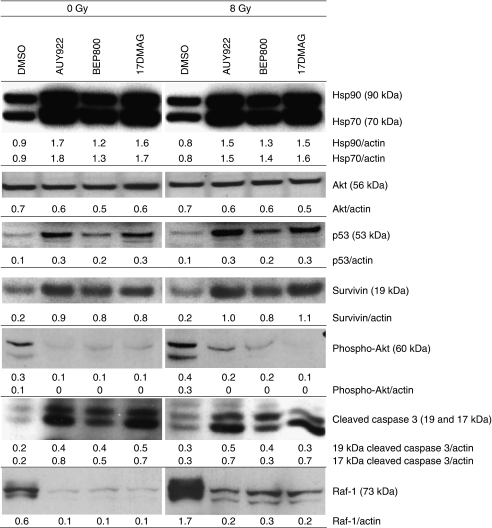
Western blot analysis of expression levels and migration patterns of Hsp90, Hsp70, survivin, Akt, p53, survivin, cleaved caspase 3, phospho-Akt and Raf-1 proteins in DMSO-treated, drug-treated and/or irradiated (8 Gy, 30 min after irradiation) HT 1080 cells. Each protein band was normalised to the intensity of *β*-actin used as loading control, and the ratios are given by the numbers.

To elucidate the molecular mechanisms of radiosensitisation (Figure 2) caused by the Hsp90 inhibitors, we further examined the expression of several proteins by western blotting (Figure 3 and Supplementary Figure S1). Figure 3 shows exemplarily western blot data of control and drug-treated HT 1080 cells probed for Hsp90, Hsp70, Akt, p53, survivin, cleaved caspase 3, Raf-1 and phospho-Akt 30 min after irradiation. As evident from the figure, the expression levels of Hsp90 and Hsp70 proteins in HT 1080 cells after drug treatment alone or in combination with IR were much higher than that in control (DMSO treated). Expression of the anti-apoptotic protein Akt in irradiated drug-treated cells was somewhat lower than those in the corresponding non-treated sample, which may be an indication of increased apoptosis. The reduction of Akt, however, did not reach statistical significance in the case of HT 1080 cells, whereas in the other tested cell lines, the level of Akt decreased significantly (Supplementary Figure S1). Similarly, Hsp90 inhibitors alone or in combination with radiation significantly suppressed the prosurvival protein Raf-1. Note that both proteins, Akt and Raf-1, are clients of Hsp90. The expression of survivin, a further anti-apoptotic and Hsp90 client protein, in drug-treated cells was higher than those in control samples.

As expected, the expression of p53, a client protein of Hsp90, varied markedly among the four tested cell lines, two of which (A549 and HT 1080) were wild type for p53, whereas GaMG and SNB19 were p53-mutated cells. Thus, control (i.e., DMSO treated) HT 1080 cells exhibited very low or no expression of p53, which is typical for p53wt cells. However, after treatment with NVP-AUY922 and 17-DMAG, and to a lesser extent in the case of NVP-BEP800, HT 1080 cells revealed detectable amounts of p53. Qualitatively similar results for the expression of Hsp90/70, p53 and survivin were obtained 24 h after irradiation (data not shown), whereas the expression of Akt was mostly recovered after treatment with all substances. At the same time, the Raf-1 protein reached a near normal level only in the case of NVP-BEP800 (data not shown).

Another effect of the Hsp90 inhibitors is an increased expression of cleaved caspase 3 in HT 1080 (Figure 3) and GaMG (Supplementary Figure S1B) cells pretreated with all tested drugs. Accordingly, the expression of phospho-Akt (Figure 3) decreased. Two other tested cell lines, A549 and SNB19, did not show any detectable changes in cleaved caspase 3 (data not shown).

To summarise, our western blot data on apoptosis-associated proteins (Figure 3 and Supplementary Figure S1) can explain the strong radiosensitising effects (Figure 2) of NVP-AUY922 and NVP-BEP800 in only two (HT 1080 and GaMG) out of four tested cell lines. Further support for the involvement of apoptosis in radiosensitising drug activity came from the measurements of cells with hypodiploid nuclei and cellular debris as indications of late-onset apoptosis, in log-scaled histograms in cell samples including both floating and adherently growing cells (Supplementary Figure S2 and Supplementary Table S1). Using this approach (Djuzenova and Flentje, 2002), we found increased fractions of cells with hypodiploid DNA content and cellular debris in three cell lines (except A549) pretreated with NVP-AUY922 and 17-DMAG (24 h after irradiation). The effect of NVP-BEP800 was less pronounced and seen only 48 h after irradiation.

In apparent contrast to the above considerations on the role of apoptosis, both NVP-AUY922 and NVP-BEP800 increased the expression of the anti-apoptotic protein survivin in irradiated HT 1080 and GaMG cells (Figure 3 and Supplementary Figure S1). This finding points towards the possibility that Hsp90 inhibition can improve the survival of a particular cell line, for instance, by conferring radioresistance on tumour cells through survivin induction. Hence, at least in the case of HT 1080 and GaMG cells, Hsp90 inhibitors seemed to simultaneously induce opposite, pro- and anti-apoptotic effects in irradiated tumour cells.

### DNA fragmentation caused by inhibitors of Hsp90 and radiation

**Figure 4:**
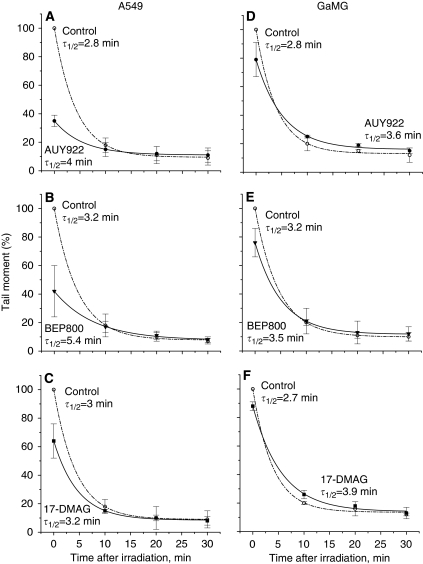

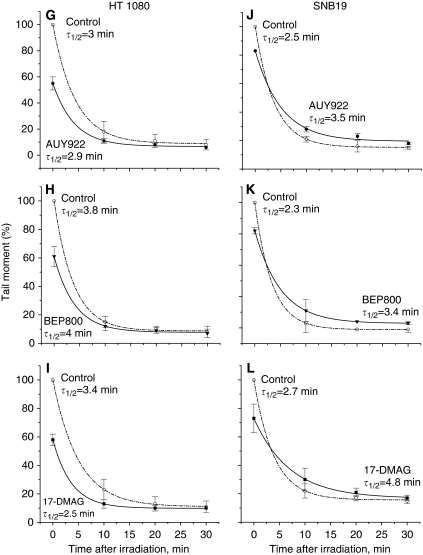
DNA damage induction and repair profiles measured by the alkaline Comet assay in human tumour cell lines (**A**–**C**, A549; **D**–**F**, GaMG; **G**–**I**, HT 1080; and **J**–**L**, SNB19) pretreated with drugs (200 nM, 24 h) and irradiated with 8 Gy of X-rays *in vitro*. The extent of DNA damage was measured quantitatively by the comet Tail Moment (TM). Each point (bar) represents the mean value (±s.d.) of TM data measured in about 150 cells from two independent experiments and normalised against untreated controls (circles, dashed line). The curves are best least-square fits of a monoexponential function to the experimental data points. Numbers indicate the repair half-time constants (min) for each curve/treatment and the ratios of the repair half-time constants of drug-pretreated cells to control cells.

To elucidate the radiosensitising effects of Hsp90 inhibitors on their colony-forming ability (Figure 2), we evaluated DNA fragmentation in control and drug-treated cells after irradiation by means of the alkaline Comet assay. The extent of DNA fragmentation was assessed from the comet TMs measured immediately (TM_0_) and up to 30 min after irradiation with 8 Gy (Figure 4).

Contrary to expectations, the three tested Hsp90 inhibitors significantly decreased the initial TM_0_ values in all cell lines studied here. Irrespective of the drug used, the initial TM_0_ values in irradiated drug-treated cells (in relation to DMSO-treated cells) reduced in the following order: A549>HT 1080>GaMG≈SNB19.

Despite the lower initial fragmentation, the restoration of DNA damage after irradiation occurred more slowly in cells pretreated with Hsp90 inhibitors. This is evident from the increased *τ*_1/2_ values given in Figure 4. The exception was the HT1080 cell line, in which the *τ*_1/2_ values were almost unaffected by the drugs.

Taken together, the data obtained by western blot (Figure 3 and Supplementary Figure S1), sub-G1 DNA measurements (Supplementary Figure S2 and Supplementary Table S1) and Comet assay (Figure 4) revealed multiple effects of Hsp90 inhibitors on tumour cells at the molecular level. Most of the effects analysed so far, however, do not account for or even disagree with the strong radiosensitising activity of these drugs revealed by the colony-forming assay (Figure 2) in all tested tumour lines. To move forward with the elucidation of the controversial data, we further analysed the impact of Hsp90 inhibitors on the induction of histone *γ*H2AX, a marker of DNA double-strand breaks (DSBs) in irradiated tumour cells.

### Effects of Hsp90 inhibitors and IR on the induction and decay of histone *γ*H2AX

**Figure 5:**
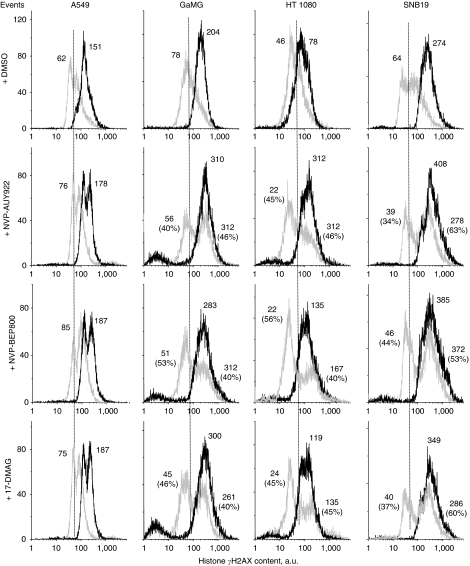
Typical distributions of nuclear histone *γ*H2AX in four tumour cell lines detected 30 min after irradiation with 8 Gy, and after a combined drug-ionising radiation (IR) treatment. Cells were stained with anti-histone *γ*H2AX and analysed by flow cytometry using logarithmic amplification mode. Numbers denote the mean histone *γ*H2AX expression for the respective cell sub-population and the percentage of cells in the sub-population. Black and light grey histograms represent irradiated and non-irradiated cells, respectively. To facilitate visual comparison, the modal *γ*H2AX values in the DMSO-treated and irradiated sample are indicated by dashed vertical lines.

**Figure 6:**
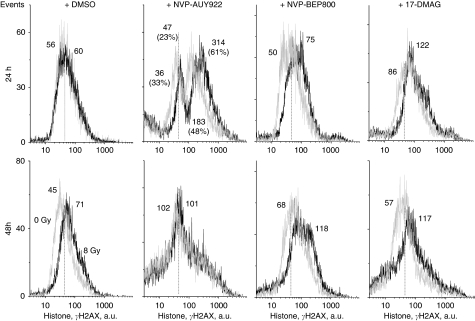
Representative distributions of nuclear histone *γ*H2AX in the GaMG tumour cell line detected 24 (upper row) and 48 h (bottom row) after irradiation with 8 Gy or after a combined drug-ionising radiation (IR) treatment. Black and light grey histograms represent irradiated and non-irradiated cells, respectively. The modal *γ*H2AX value in the control (DMSO treated) non-irradiated sample is indicated by a dashed vertical line. For details, see legend to Figure 5.

The induction of DNA DSBs, as analysed by the expression of phosphorylated histone H2AX (Rogakou *et al*, 1998), was measured 30 min (Figure 5), and 24 and 48 h (Figure 6) after irradiation of tumour cells, non-treated or pretreated with Hsp90 inhibitors. As evident from the flow cytograms of DMSO-treated control cultures (Figure 5, upper row, light grey histograms), the background expression of histone *γ*H2AX differed markedly among the four tested cell lines. HT 1080 cells exhibited the lowest background level of *γ*H2AX with the mean fluorescence intensity of ∼46 a.u. In A549, SNB19 and GaMG cells, the amounts of endogenous histone *γ*H2AX were about 62, 64 and 78 a.u., respectively. At 30 min after IR, the expression of histone *γ*H2AX in control cells increased by a factor of 2–4 (Figure 5, upper row, black histograms).

In the majority (except A549) of cell lines tested, Hsp90 inhibitors induced dramatic cell type-specific changes in *γ*H2AX expression (Figure 5, rows 2–4, light grey histograms), compared with DMSO-treated controls (Figure 5, upper row, light grey histograms). The *γ*H2AX histograms of drug-treated cells were mostly bimodal and spread over 2–3 decades of fluorescence intensity. This finding implies that each cell line consists of two distinct sub-populations differing strongly in their sensitivity to Hsp90 inhibitors.

Combined drug-IR treatment (Figure 5, rows 2–4, black histograms) strongly increased *γ*H2AX expression, compared with each treatment modality alone. In three out of four cell lines, combined treatment produced very narrow and mostly unimodal distributions of histone *γ*H2AX, which contrasted with those induced by drugs alone (Figure 5, rows 2–4, light grey histograms). The exception was the lung carcinoma line (A549), in which the combined drug-IR treatment caused a bimodal expression pattern of *γ*H2AX (Figure 5, left-hand column, black histograms), similar to that caused by drug treatment alone. Besides this, the amounts of DNA DSBs in A549 cells after combined drug-IR treatment increased only moderately (by about 20%) above the corresponding data of irradiated cell samples without Hsp90 inhibitors.

In all tested cell lines, increasing the repair time from 30 min to 24 and 48 h after *IR alone* resulted in a near-complete restoration of the expression of histone *γ*H2AX to the background level (Figure 6, only data for GaMG cells are shown). Drug-treated and then irradiated cells, however, still exhibited elevated amounts of histone *γ*H2AX 24 h after irradiation (Figure 6, upper row, light grey histograms). At 48 h after irradiation, the amounts of residual histone *γ*H2AX further decreased, but the values were still higher (Figure 6, bottom row, black histograms) than those in the corresponding control sample (Figure 6, bottom row, left-hand light grey histogram). Qualitatively similar data were obtained for the other three tested cell lines (averaged data from three independent experiments with four cell lines are shown in Supplementary Figure S3).

### Effects of Hsp90 inhibitors and IR on cell-cycle progression

**Figure 7:**
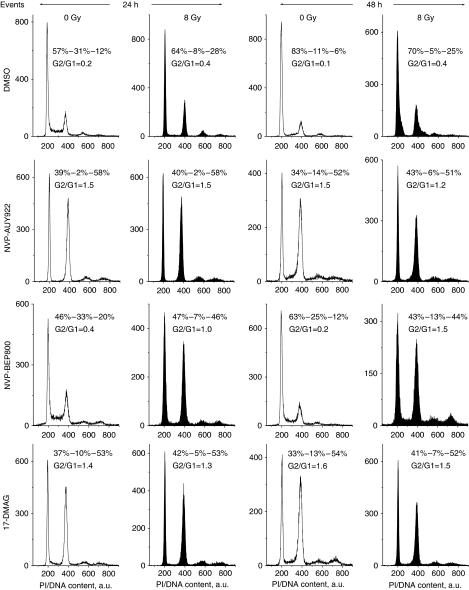
Effects of Hsp90 inhibitors, ionising radiation (IR) and combined drug-IR treatment on the cell cycle-phase distribution in the A549 cell line. Cells were treated with drugs and/or irradiated with 8 Gy, cultured for 24 or 48 h, fixed, permeabilised, stained with propidium iodide (PI) and analysed for DNA content by flow cytometry using linear signal amplification. DNA histograms were deconvoluted with ModFit Software. Numbers denote the percentage of cells in G1, S and G2/M phases and G2/G1 ratios in each cell sample. Filled and unfilled histograms represent irradiated and non-irradiated cells, respectively.

Further efforts to identify the mechanisms underlying the radiosensitising effect of Hsp90 inhibitors were focused on their possible impact on cell-cycle progression. Cells were treated with 200 nM of drugs for 24 h and analysed by flow cytometry for the cell cycle-phase distribution. As seen from Supplementary Table S2, Hsp90 inhibitors caused a depletion of the S phase and an accumulation of cells with G2/M DNA content. Drug-treated cells were then transferred into drug-free medium, irradiated with 8 Gy, cultured for the next 24 and 48 h and then analysed once again for cell-cycle distribution. Because of space limitation, representative cell-cycle data are provided only for A549 cells (Figure 7), whereas histograms for the other three cell lines are shown in Supplementary Figure S4. Supplementary Table S3 summarises cell-cycle data from three independent experiments for all cell lines tested. The large portions of cells in S and G2/M phases in the untreated control sample (Figure 7, top row, left-hand histogram) prove that, at the beginning of these experiments, the cell culture was in the exponential growth phase.

In non-irradiated samples (first and third columns in Figure 7), NVP-AUY922 and 17-DMAG induced a marked long-term increase in the G2/M peak, lasting for at least 48 h after drug removal. Both drugs also caused a strong depletion of the S phase during the first 24 h, followed by partial recovery during the subsequent incubation for up to 48 h in drug-free medium (Figure 7, third column). In this particular cell line, treatment with NVP-BEP800 alone caused comparatively small changes (G2/G1=0.4) in cell-cycle distribution, which were partly recovered 48 h after incubation in drug-free medium (G2/G1=0.2).

As expected, radiation alone caused a significant increase in G2/M cells (Figure 7, top row, second and fourth histograms from the left). In the case of NVP-AUY922 and 17-DMAG, combined drug-IR treatment did not cause any additional changes in cell-cycle distribution, compared with drug treatment alone. In sharp contrast, combined NVP-BEP800-IR treatment (Figure 7, third row) resulted in a much stronger cell-cycle disturbance than each agent alone.

### Effects of Hsp90 inhibitors on the expression of cell cycle-related proteins

**Figure 8:**
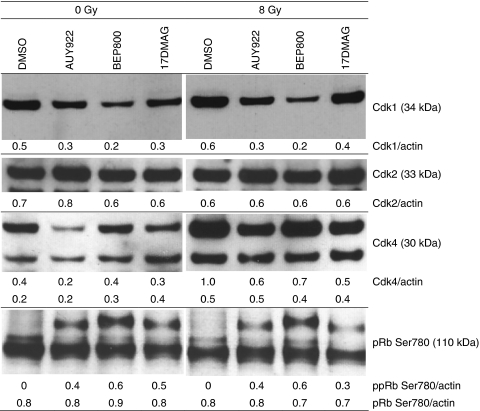
Effects of Hsp90 inhibitors on the expression of cell-cycle regulatory proteins in the SNB19 tumour cell line. Total cell extracts were prepared 30 min after irradiation with 8 Gy, resolved by SDS–PAGE, blotted and immunostained according to standard procedures. Each protein band was normalised to the intensity of *β*-actin used as loading control, and the ratios are denoted by the numbers.

The observed alterations in the cell cycle caused by Hsp90 inhibitors prompted us to analyse the expression levels of various cell cycle-regulating factors, such as cyclin-dependent kinases (Cdk1, Cdk2, Cdk4) and pRb, by western blotting. As shown in Figure 8 and Supplementary Figure S5, Hsp90 inhibitors reduced the levels of Cdk1 in all tested cell lines, although to different extents. Similarly, the levels of Cdk4 decreased significantly in case of NVP-AUY922 and 17-DMAG, and to a lesser extent in the case of NVP-BEP800. The expression of phosphorylated Rb decreased strongly in two (A549 and HT 1080) out of four tested cell lines after Hsp90 inhibition with all tested substances. Another finding was that Cdk2, a close relative of the Hsp90-dependent Cdk4 kinase, was unaffected by drug treatment.

## Discussion

Previous studies have shown that inhibition of Hsp90 enhances the radiation response of several cell lines derived from a variety of human tumour entities (Bisht *et al*, 2003; Enmon *et al*, 2003; Machida *et al*, 2003; Russell *et al*, 2003; Bull *et al*, 2004; Harashima *et al*, 2005; Dote *et al*, 2006). These findings validate the molecular chaperone Hsp90 as a clinically relevant target for tumour radiosensitisation. The molecular mechanisms underlying the interaction between IR and conventional Hsp90 inhibitors, such as the geldanamycin derivatives 17-AAG and 17-DMAG, have not yet been clearly identified. One of the proposed mechanisms to explain the radiosensitising effects of geldanamycins involves the selective degradation of several key proteins responsible for radioresistance, including ErbB2, EGFR, Raf-1 and Akt (for review, see Chinnaiyan *et al*, 2006). However, the degradation of ErbB2 induced either by 17-DMAG (Bull *et al*, 2004; Dote *et al*, 2005) or by siRNA (Dote *et al*, 2005) does not enhance the radiosensitivity of various carcinoma cell lines. These findings suggest the involvement of other mechanisms in the radiosensitising activity of Hsp90 inhibitors. Besides this, geldanamycin and its derivatives have several limitations for clinical use (see the Introduction section, Kelland *et al*, 1999; Eiseman *et al*, 2005; Eccles *et al*, 2008).

In contrast to geldanamycin derivatives, the isoxazole resorcinol Hsp90 inhibitor NVP-AUY922 has recently shown promising results with regard to its pharmaceutical and pharmacological properties, in conjunction with a well-tolerable toxicity against different tumour cell types *in vitro* and *in vivo* (Eccles *et al*, 2008; Jensen *et al*, 2008). Compared with NVP-AUY922, the novel, structurally distinct Hsp90 inhibitor NVP-BEP800 tested here has an improved oral bioavailability (Brough *et al*, 2009; Massey *et al*, 2010). In this study, we systematically applied a multitarget approach to explore the impact of NVP-AUY922 and NVP-BEP800 on the radiation response of tumour cells.

Our colony survival experiments identified NVP-AUY922 and NVP-BEP800 as potent radiosensitisers in all tumour cell lines studied here (Figure 2). However, only two (HT 1080 and GaMG) out of four tested tumour cell lines exhibited, after treatment with NVP-AUY922 (but not with NVP-BEP800), a distinct expression of cleaved caspase 3, as revealed by western blot analysis (Figure 3 and Supplementary Figure S1). At the same time, the levels of Raf-1, and to a lesser extent of Akt, were reduced by the Hsp90 inhibitors in all tested cell lines. The two proteins (cleaved caspase 3 and Raf-1) are of particular interest because their inhibition has been associated with enhanced radiation sensitivity in some systems (Gupta *et al*, 2001; Shintani *et al*, 2006). The role of apoptosis in the radiosensitisation with the novel Hsp90 inhibitors was further supported by the increased percentage of cells with hypodiploid DNA contents and debris (Supplementary Figure S2). This approach revealed the late onset of apoptosis in most cell lines (except A549) pretreated with NVP-AUY922 and 17-DMAG, and to a much lesser extent after treatment with NVP-BEP800 (Supplementary Table S1 and Supplementary Figure S2). Consequently, the radiosensitising activities of NVP-AUY922 and NVP-BEP800 in all tested cell lines cannot be explained solely by the drug-mediated susceptibility to apoptosis.

Functional tumour suppressor protein p53 was apparently not essential for the radiosensitising action of NVP-AUY922 and NVP-BEP800, because both drugs radiosensitised all tested cell lines, independent of their p53 status (Figures 2 and 3). This finding is consistent with the recent data for two non-small-cell lung cancer cell lines, NCI-H460 and A549 (Koll *et al*, 2008), but it conflicts with the results for squamous carcinoma cell lines (Shintani *et al*, 2006), indicating that the Hsp90 inhibitor 17-AAG is a more efficient radiosensitiser in a cell line with p53 wild type compared with four p53-mutated cell lines.

Summarising the western blot data shown in Figure 3, neither changes in survival markers (Hsp90, Hsp70, Akt, phospho-Akt, Raf-1 and survivin) and apoptosis-associated protein (cleaved caspase 3) nor alterations in p53 were significant to account for the sensitivity of two (A549 and SNB19) out of four tested cell lines to NVP-AUY922 and NVP-BEP800, either as a drug treatment alone or in combination with radiation.

At variance with expectations, the alkaline Comet assay revealed, in all tested cell lines, a decrease in TM values and thus a lower DNA fragmentation after combined drug-IR treatment, compared with those induced by IR alone (Figure 4). The minor DNA fragmentation can be explained by the remarkable changes in the cell cycle caused by Hsp90 inhibitors, that is, an S-phase depletion and G2/M arrest (Figure 7), which were apparently associated with large alterations in DNA compactness. As shown elsewhere (Olive and Banáth, 1993), cells in the S phase show the highest TM values, whereas the TM values of G2/M cells are even lower than those in the G1 phase.

It should be noted that the Comet assay does not provide a measure for radiosensitivity in the conventional sense, that is, chromosome breakage, micronucleus formation, reduced growth and cloning survival, or increased mutation frequency. Rather, the Comet assay evaluates chromatin integrity as a function of time immediately after irradiation. Therefore, differences in chromatin compaction can strongly affect the results of the Comet assay (Noz *et al*, 1996). The recognition of DNA damage by the Comet assay is also well known to rely on a number of factors involved in the release of DNA from the nuclear protein matrix (Speit and Hartmann, 1999). In view of the above considerations, the observed drug-mediated reduction of IR-induced DNA fragmentation (Figure 4) might have resulted from the drug-mediated, cell cycle-related changes in the compactness of chromatin/DNA structure.

Despite the lower initial DNA fragmentation detected by the Comet assay, the rates of DNA restitution in three cell lines (A549, GaMG and SNB19) after a combined drug-IR treatment were lower than those after IR alone. These results strongly suggest the role of Hsp90 and its clients in the restitution of IR-induced DNA fragmentation. This conclusion is consistent with recent findings that combined 17-DMAG/IR treatment inhibits DNA repair in two human pancreatic cell lines, analysed by a neutral Comet assay (Dote *et al*, 2005). Similarly, an alkaline Comet assay has also revealed an impaired radiation-induced DNA repair in DMAG-treated lung carcinoma H460 cells (Koll *et al*, 2008). Contrary to our data, Koll *et al* (2008) have also found increased TM values after irradiation of DMAG-treated cells, compared with non-treated ones. This discrepancy can be explained by the differences in the experimental protocols, including cell scraping in ice-cold PBS, cell lines used and so on.

A further critical determinant of radiation-induced cell death is the induction and repair of DNA DSBs, which can be probed very sensitively by histone *γ*H2AX (Rogakou *et al*, 1998). In this study, drug-treated tumour cell samples were found to express two distinct sub-populations (responding and non-responding to drug) differing markedly in their *γ*H2AX contents (Figure 5) spreading over 2–3 decades of intensity, as well as in the percentage of cells in each sub-population. Given that all cell lines used here had similar cell-cycle distributions before drug treatment, the *γ*H2AX expression mediated by the drugs alone (without IR) was more cell-line specific rather than coupled with the cell cycle.

Combined drug-IR treatment induced higher amounts of DNA DSBs measured by histone *γ*H2AX than each treatment alone (Figure 5). Moreover, the repair of DNA DSBs induced by combined treatment occurred much more slowly than after irradiation alone (Figure 6 and Supplementary Figure S3). These data are in accordance with the delayed dispersal of histone *γ*H2AX in the MiaPaCa pancreas carcinoma cell line, which received the combined 17DMAG/radiation treatment (Dote *et al*, 2006). The authors suggest that 17DMAG inhibits the repair of DNA DSBs induced by radiation (Dote *et al*, 2006), Similarly, an inhibition of homologous DNA recombination repair, that is, degradation of BRCA2 and alteration of Rad51 by 17-AAG, causes the radiosensitisation of prostate carcinoma DU145 and lung squamous carcinoma SQ-5 cell lines (Noguchi *et al*, 2006). Similar effects on histone *γ*H2AX, for example, prolonged persistence of DNA damage measured by this sensitive marker, have been shown in several studies using HDAC inhibitors that indirectly block Hsp90 by acetylation (Zhang *et al*, 2007; Zuco *et al*, 2010). As suggested by a reviewer, we analysed the expression of several DNA repair proteins, including Ku70, Ku80, Rad50, Rad51, DNA-PKcs and BRCA2. We found that all drug-treated cells were depleted of Ku70/80 proteins (data not shown), whereas other proteins were not significantly affected by drug treatment. Further studies will be needed to clarify the mechanisms of DNA repair distortion, which will be a subject of future research in our laboratory.

Finally, all tested Hsp90 inhibitors caused a substantial G2/M block that was even more pronounced after subsequent irradiation in case of NVP-BEP800-treated cells. In addition, NVP-AUY922 induced a temporary depletion of S-phase cells. These data are in agreement with the ability of 17-DMAG and NVP-AUY922 to cause a loss of S phase and an accumulation of cells with G2/M DNA content (Eccles *et al*, 2008; Koll *et al*, 2008). The effects of Hsp90 inhibitors on the cell cycle reported here and elsewhere (Eccles *et al*, 2008; Koll *et al*, 2008) are, however, quite contrary to the findings that 17-DMAG abrogates the radiation-induced arrest of three human tumour cell lines in the S and G2 phases (Bull *et al*, 2004). Similarly, geldanamycin has also been found to abolish G2-phase arrest in human colon adenocarcinoma cells that are null or mutant for p53 (Moran *et al*, 2008).

To explain remarkable cell-cycle changes in response to Hsp90 inhibitors, we analysed the expression levels of several cell cycle-dependent proteins. It is worth mentioning that important proteins related to the cell cycle, including Cdk1, Cdk2, Cdk4 and p53 (Figure 8), are well-known clients of Hsp90 (Picard, 2002). We found that Hsp90 inhibition led to downregulation of Cdk4 in all tested cell lines. However, only two cell lines, A549 and HT 1080 (both wt p53), exhibited hypophosphorylation of Rb, which functions as a blocker of cell-cycle progression at the G1/S checkpoint (Stewart *et al*, 2003). Another finding is that Hsp90 inhibitors markedly reduced Cdk1 levels in HT 1080, GaMG and SNB19, and to a lesser extent in A549 cells, thus causing a G2/M arrest that is independent of the cellular p53 status. Checkpoint protein Cdk1 has been identified as an Hsp90 client and is a key transducer of G2/M-phase arrest in response to the drug treatment.

To sum up, our data demonstrate enhanced radiosensitivity in four solid tumour cell lines pretreated with NVP-AUY922 or NVP-BEP800. The complex mechanisms underlying the radiosensitisation by these novel Hsp90 inhibitors involve apparently multiple, cell-line-specific pathways that lead to the destabilisation and degradation of several Hsp90 client proteins, thus causing a dramatic cell-cycle impairment that leads to a slower proliferation of tumour cells, increased DNA damage and protraction of DNA repair after irradiation, and to a lesser extent, to apoptosis. The data are of particular interest for the radiation therapy of cancer, because NVP-AUY922 is currently in clinical trials Phase I–II (www.clinicaltrials.gov). Besides raising important questions with regard to the mechanisms of radiosensitisation, the *in vitro* data presented here will surely prompt further clinical studies on the possibility of combining NVP-AUY922 and NVP-BEP800 with radiation, which may open up a promising approach for improved local control of cancer.

## Figures and Tables

**Table 1:** Cloning efficiencies and radiosensitivity parameters^a^ of *in vitro* irradiated tumor cell lines untreated and pretreated with the Hsp90 inhibitors

**Cell line**	**Plating efficiency**	**SF2**	***α* (Gy^−1^)**	***β* (Gy^−2^)**	**D_10_ (Gy)^b^**	**Factor (D_10_ control)/(D_10_+inh.)**
*A549 – contr.*	0.8±0.1	0.70±0.05	0.1±0.03	0.04±0.01	6	1.0
+AUY922	0.5±0.01	0.33±0.06	0.4±0.2	0.04±0.05	4	1.5
+BEP800	0.8±0.1	0.52±0.1	0.2±0.1	0.04±0.01	5	1.2
+17DMAG	0.7±0.1	0.41±0.08	0.3±0.2	0.07±0.05	4	1.5
						
*GaMG – contr.*	0.3±0.1	0.84±0.01	0.01±0.01	0.04±0	8	1.0
+AUY922	0.1±0	0.46±0.04	0.1±0.07	0.1±0	4	1.8
+BEP800	0.3±0.1	0.57±0.08	0.1±0.1	0.07±0.01	5	1.4
+17DMAG	0.1±0.05	0.44±0.02	0.1±0	0.1±0	4	1.8
						
*HT1080 – contr.*	0.1±0.04	0.81±0	—	0.05±0	7	1.0
+AUY922	0.02±0.03	0.63±0.2	0.1±0.2	0.06±0.01	5	1.4
+BEP800	0.1±0.03	0.59±0.2	0.06±0.08	0.08±0.03	5	1.4
+17DMAG	0.03±0.01	0.70±0.1	—	0.1±0.02	5	1.4
						
*SNB19 – contr.*	0.3±0.1	0.53±0.3	0.3±0.4	0.03±0.04	6	1.0
+AUY922	0.2±0.1	0.40±0.3	0.4±0.4	0.07±0.04	5	1.3
+BEP800	0.2±0.1	0.45±0.2	0.3±0.2	0.06±0.01	5	1.3
+17DMAG	0.1±0.1	0.53±0.2	0.1±0.1	0.1±0	4	1.3

Abbreviations: contr.=control; inh.=inhibitor.

a Mean (±s.d.) from at least two independent experiments.

b D_10_ is the radiation dose required to reduce clonogenic survival by 10%.
