# Supplementary material for: Novel HSP90 inhibitors, NVP-AUY922 and NVP-BEP800, radiosensitise tumour cells through cell-cycle impairment, increased DNA damage and repair protraction
Source: Br J Cancer. 2010 May 25;102(11):1578–91. doi: 10.1038/sj.bjc.6605683 (PMC2883148; doi:10.1038/sj.bjc.6605683)
Supplement: Supplementary Information [file 6605683x1.doc]

Stingl et al.

**Supplementary Information**

**Antibodies**

Primary antibodies used were: mouse monoclonal anti-phospho-histone H2AX FITC-conjugate (Millipore, Schwalbach, Germany), rabbit monoclonal anti-(cleaved)-caspase-3 (Cell Signaling, Danvers, MA, USA), rabbit polyclonal anti-Akt and mouse monoclonal anti-phospho-Akt (Cell Signaling, Danvers, MA, USA), rabbit polyclonal anti-survivin (R&D Systems, Minneapolis, MN, USA), mouse monoclonal anti-p53 (Calbiochem, [Merck KGaA](http://www.merck.de/), Darmstadt, Germany), mouse monoclonal anti-Hsp70 and mouse monoclonal anti-Hsp90 (BD Pharmingen, Heidelberg, Germany), mouse monoclonal anti-cleaved PARP (Cell Signaling, Danvers, MA, USA), mouse monoclonal anti-cdc2 (Cell Signaling, Danvers, MA, USA), rabbit polyclonal anti-phospho-Rb (Cell Signaling, Danvers, MA, USA), rabbit polyclonal anti-cdk2 (Millipore, Temecula, CA, USA), rabbit polyclonal anti-DNA-PK (Cell Signaling, Danvers, MA, USA), rabbit polyclonal anti-Ku80 (Cell Signaling, Danvers, MA, USA), rabbit polyclonal anti-Ku70 (Cell Signaling, Danvers, MA, USA), rabbit polyclonal anti-Raf-1 (Santa Cruz Biotechnology, Santa Cruz, CA, USA), rabbit polyclonal anti-Cdk4 (Santa Cruz Biotechnology, Santa Cruz, CA, USA), mouse monoclonal anti-actin (Sigma, Deisenhofen, Germany). Secondary species-specific antibodies for Western blot were labeled with horseradish-peroxidase (DAKO, Hamburg, Germany).

**Figure legends**

**Figure S1.** Western blot analysis of expression levels and migration patterns of Hsp90, Hsp70, Akt, p53, survivin, cleaved caspase 3, phospho-Akt and Raf-1 proteins, in DMSO-treated, drug-treated and/or irradiated (8 Gy, 30 min post-irradiation) A549 (part ***A***), GaMG (part ***B***) and SNB19 (part ***C***) cell lines. Each protein band was normalized to the intensity of β-actin used as loading control, and the ratios are depicted as numbers. Cleaved caspase 3 was not detectable in A549 and SNB19 cells.

**Figure S2.** Percentage of cells with hypodiploid DNA content and cellular debris in drug-treated and irradiated tumor cell lines (***A*** – A549, ***B*** – GaMG, ***C*** – HT 1080, ***D*** – SNB19), measured flow cytometrically. Drug treated and irradiated (8 Gy) cells were cultivated for 24 and 48 h. At indicated time intervals the cells were detached with trypsin, treated with saponin and RNAse, stained with PI and then analyzed for red fluorescence by flow cytometry. The samples include both floating and trypsinized cells. PI/DNA fluorescence was acquired in the logarithmic mode. The numbers represent the percentages of events for hypodiploid nuclei and debris in non-irradiated and irradiated cell samples, computed by means of the WinMDI Software.

**Figure** **S3**. Kinetics of disappearance of nuclear histone γH2AX in drug-treated tumor cell lines detected 30 min, 24 and 48 h post-irradiation with 8 Gy. The values (mean ± SD) are averaged for 3 independent experiments. To facilitate visual comparison, the modal γH2AX value in DMSO treated and irradiated sample is set to unity throughout the experiments.

**Figure S4.** Effects of the Hsp90 inhibitors, IR and combined drug-IR treatment on the cell cycle-phase distribution in GaMG (part ***A***), HT 1080 (part ***B***) and SNB19 (part ***C***) cell lines. Drug-treated and non-treated cells were irradiated with 8 Gy, cultured for 24 and 48 h, fixed, permeabilized, stained with PI, and analyzed for DNA content by flow cytometry using linear signal amplification. Deconvolution of DNA histograms was performed with ModFit Software. The numbers denote the percentage of cells in G1-, S- and G2/M phases and G2/G1 ratios in each cell sample. Arrows show the fraction of hyperdiploid cells. Filled and unfilled histograms represent irradiated and non-irradiated cells, respectively.

**Figure S5.** Effects of Hsp90 inhibitors on the expression of cell-cycle regulatory proteins in A549, HT 1080 and SNB19 cells (parts ***A***, ***B*** and ***C***, respectively). Total cell extracts were prepared 30 min after irradiation with 8 Gy, resolved by SDS-PAGE, blotted and immunostained according standard procedure.

**Table S1**

Percentage of cells with hypodiploid DNA content and cellular debris after treatment (200 nM, 24 h) with different Hsp90 inhibitors followed by irradiation (8 Gy) and subsequent incubation for 24 and 48 h

# Table S2

Cell cycle-phase distribution in 4 tumor cell lines after drug treatment (200 nM, 24 h)

**Table S3**

Cell cycle-phase distribution in 4 tumor cell lines pretreated with different Hsp90 inhibitors detected 24 h and 48 h post-irradiation (8 Gy)

Stingl et al. Supplementary Information

| **0 Gy** | | | | | | | **8 Gy** | | | | |  |
| --- | --- | --- | --- | --- | --- | --- | --- | --- | --- | --- | --- | --- |
| **DMSO** | **AUY922** | | **BEP800** | | **17DMAG** | | **DMSO** | **AUY922** | **BEP800** | **17DMAG** | |  |
| 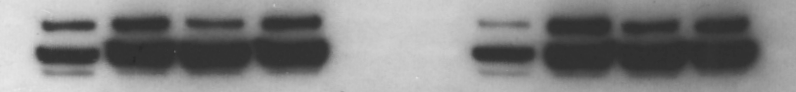 | | | | | | | 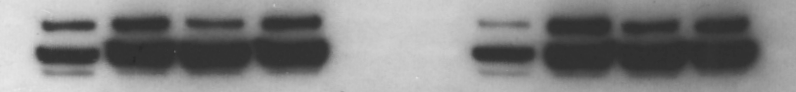 | | | | | Hsp90 (90 kDa) |
|  |  |  |  |  |  |  |  |  |  |  |  | Hsp70 (70 kDa) |
| 0.4 | 0.9 | | 0.5 | | 0.8 | | 0.3 | 1.1 | 0.6 | 0.8 | | Hsp90/actin |
| 0.9 | 1.8 | | 1.6 | | 1.6 | | 0.9 | 1.7 | 1.7 | 1.6 | | Hsp70/actin |
| 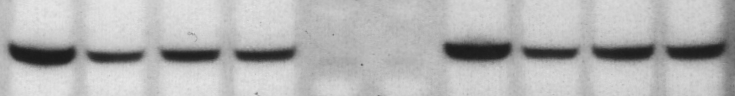 | | | | | | | 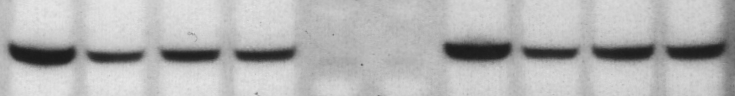 | | | | | Akt (56 kDa) |
| 1.0 | 0.6 | | 0.7 | | 0.7 | | 1.1 | 0.6 | 0.7 | 0.8 | | Akt/actin |
| 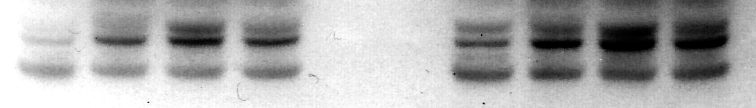 | | | | | | | 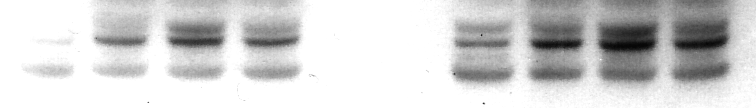 | | | | | p53 (53 kDa) |
| 0.1 | 0.3 | | 0.3 | | 0.3 | | 0.3 | 0.4 | 0.4 | | 0.4 | p53/actin |
| 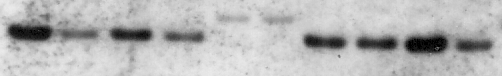 | | | | | | | 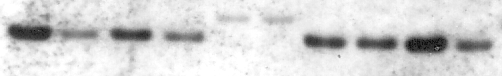 | | | | | survivin (19 kDa) |
| 1.4 | 0.8 | | 1.3 | | 0.8 | | 1.3 | 0.7 | 1.2 | | 0.8 | survivin/actin |
| 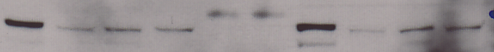 | | | | | | | 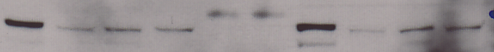 | | | | | phospho-Akt (60 kDa) |
| 0.4 | 0.1 | | 0.1 | | 0.1 | | 0.4 | 0.1 | 0.2 | | 0.2 | phospho-Akt/actin |
| 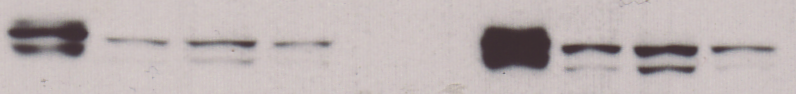 | | | | | | | 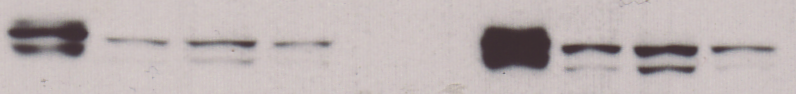 | | | | | Raf-1 (73 kDa) |
| 1.2 | | 0.1 | | 0.2 | | 0.1 | 1.7 | 0.3 | 0.7 | | 0.2 | Raf-1/actin |

**Figure S1A.** Western blot of Hsp90, Hsp70, Akt, p53, survivin, phospho-Akt and Raf-1 proteins in DMSO-treated, drug-treated and/or irradiated (8 Gy, 30 min post-irradiation) A549 cells. Each protein band was normalized to the intensity of β-actin used as loading control, and the ratios are depicted as numbers. Cleaved caspase 3 was not detectable.

Stingl et al. Supplementary Information

| **0 Gy** | | | | | | | **8 Gy** | | | | |  |
| --- | --- | --- | --- | --- | --- | --- | --- | --- | --- | --- | --- | --- |
| **DMSO** | **AUY922** | | **BEP800** | | **17DMAG** | | **DMSO** | **AUY922** | **BEP800** | **17DMAG** | |  |
| 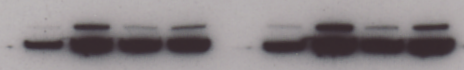 | | | | | | | 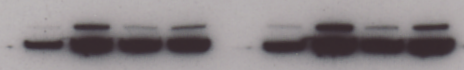 | | | | | Hsp90 (90 kDa) |
|  |  |  |  |  |  |  |  |  |  |  |  | Hsp70 (70 kDa) |
| 0.1 | 0.4 | | 0.2 | | 0.3 | | 0.2 | 0.5 | 0.2 | 0.4 | | Hsp90/actin |
| 0.5 | 1.5 | | 1.3 | | 1.5 | | 0.8 | 1.4 | 1.2 | 1.4 | | Hsp70/actin |
| 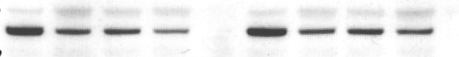 | | | | | | | 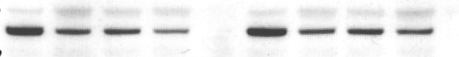 | | | | | Akt (56 kDa) |
| 0.5 | 0.4 | | 0.4 | | 0.2 | | 0.6 | 0.3 | 0.4 | 0.4 | | Akt/actin |
| 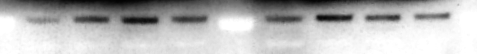 | | | | | | | 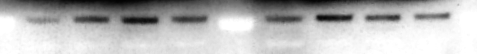 | | | | | p53 (53 kDa) |
| 0.1 | 0.3 | | 0.4 | | 0.2 | | 0.3 | 0.4 | 0.3 | | 0.3 | p53/actin |
| 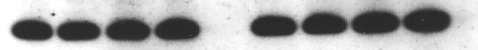 | | | | | | | 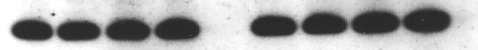 | | | | | survivin (19 kDa) |
| 1.9 | 2.1 | | 2.1 | | 2.1 | | 1.9 | 1.9 | 2.0 | | 2.3 | survivin/actin |
| 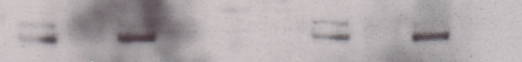 | | | | | | | 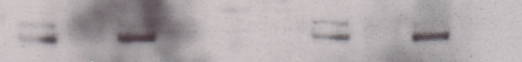 | | | | | phospho-Akt (60 kDa) |
| 0.1 | 0 | | 0.2 | | 0 | | 0.1 | 0 | 0.2 | | 0 | phospho-Akt/actin |
| 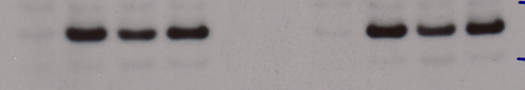 | | | | | | | 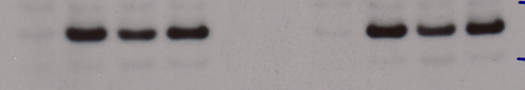 | | | | | cleaved caspase 3 (19 kDa) |
| 0 | 0.7 | | 0.5 | | 0.6 | | 0 | 0.5 | 0.4 | | 0.5 | cleaved caspase 3 /actin |
| 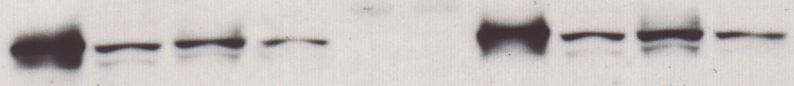 | | | | | | | 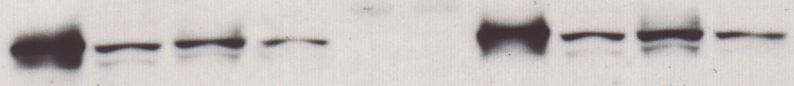 | | | | | Raf-1 (73 kDa) |
| 1.0 | | 0.2 | | 0.3 | | 0.1 | 1.2 | 0.2 | 0.5 | | 0.2 | Raf-1/actin |

**Figure S1B.** Western blot of Hsp90, Hsp70, Akt, p53, survivin, cleaved caspase 3, phospho-Akt and Raf-1 proteins in DMSO-treated, drug-treated and/or irradiated (8 Gy, 30 min post-irradiation) GaMG cell line. Each protein band was normalized to the intensity of β-actin used as loading control, and the ratios are depicted as numbers.

Stingl et al. Supplementary Information

| **0 Gy** | | | | | | | **8 Gy** | | | | |  |
| --- | --- | --- | --- | --- | --- | --- | --- | --- | --- | --- | --- | --- |
| **DMSO** | **AUY922** | | **BEP800** | | **17DMAG** | | **DMSO** | **AUY922** | **BEP800** | **17DMAG** | |  |
| 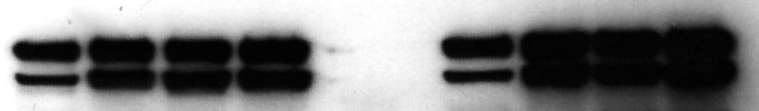 | | | | | | | 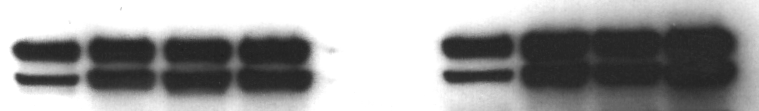 | | | | | Hsp90 (90 kDa) |
|  |  |  |  |  |  |  |  |  |  |  |  | Hsp70 (70 kDa) |
| 1.2 | 1.5 | | 1.4 | | 1.6 | | 1.4 | 1.6 | 1.4 | 1.6 | | Hsp90/actin |
| 0.6 | 1.2 | | 1.5 | | 1.5 | | 0.7 | 1.3 | 1.2 | 1.6 | | Hsp70/actin |
| 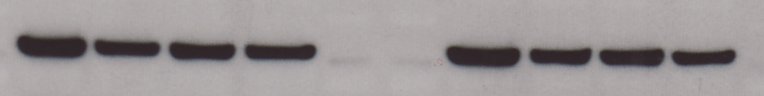 | | | | | | | 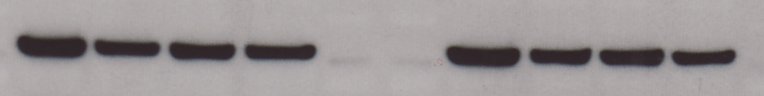 | | | | | Akt (56 kDa) |
| 1.3 | 1.0 | | 1.1 | | 1.0 | | 1.3 | 0.8 | 0.9 | 0.8 | | Akt/actin |
| 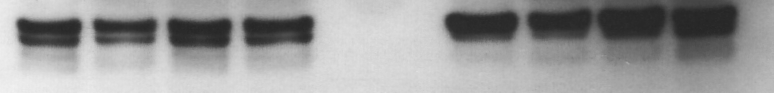 | | | | | | | 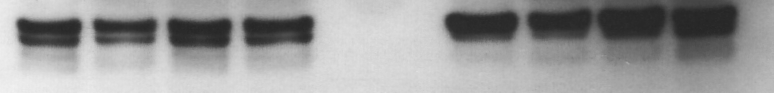 | | | | | p53 (53 kDa) |
| 1.1 | 0.9 | | 1.2 | | 1.1 | | 1.1 | 0.8 | 1.3 | | 1.2 | p53/actin |
| 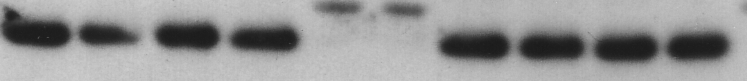 | | | | | | | 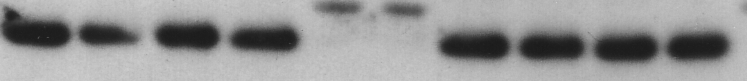 | | | | | survivin (19 kDa) |
| 2.4 | 2.3 | | 2.4 | | 2.5 | | 2.4 | 2.3 | 2.4 | | 2.4 | survivin/actin |
| 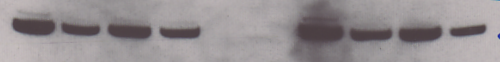 | | | | | | | 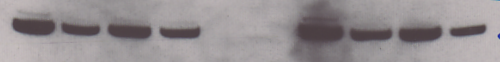 | | | | | phospho-Akt (60 kDa) |
| 0.7 | 0.5 | | 0.6 | | 0.5 | | 0.7 | 0.5 | 0.6 | | 0.4 | phospho-Akt/actin |
| 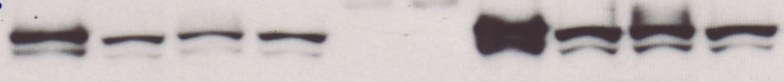 | | | | | | | 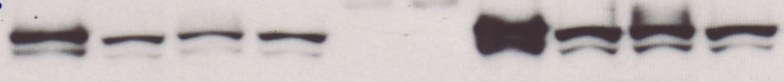 | | | | | Raf-1 (73 kDa) |
| 0.4 | | 0.1 | | 0.1 | | 0.2 | 0.9 | 0.3 | 0.4 | | 0.3 | Raf-1/actin |

**Figure S1C.** Western blot analysis of Hsp90, Hsp70, Akt, p53, survivin, phospho-Akt and Raf-1 proteins in DMSO-treated, drug-treated and/or irradiated (8 Gy, 30 min post-irradiation) SNB19 cell line. Each protein band was normalized to the intensity of β-actin used as loading control, and the ratios are depicted as numbers. Cleaved caspase 3 was not detectable.

Stingl et al. Supplementary Information

|  | 24 h | | 48 h | |
| --- | --- | --- | --- | --- |
|  | **0 Gy** | **8 Gy** | **0 Gy** | **8 Gy** |
| DMSO | 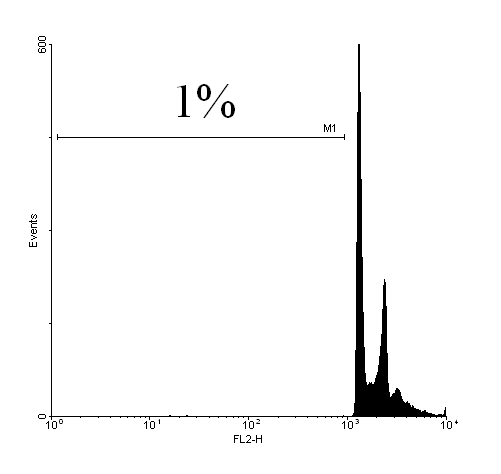 | 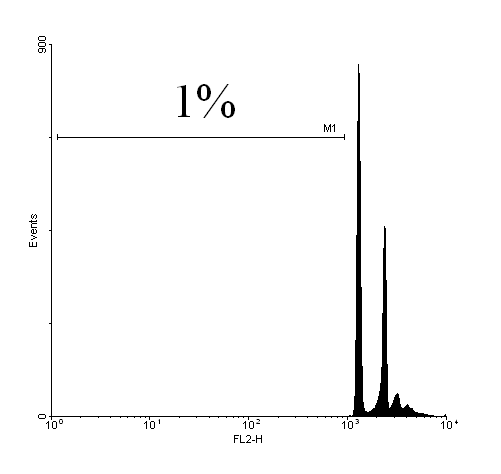 | 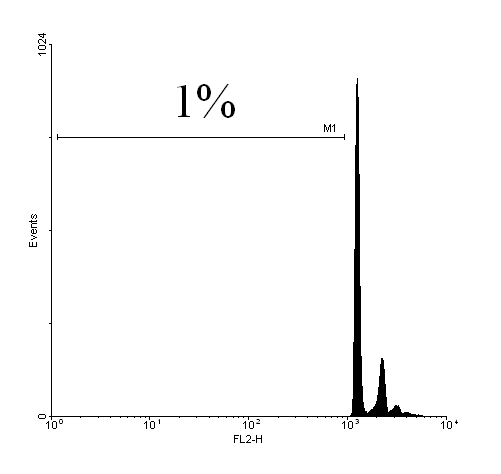 | 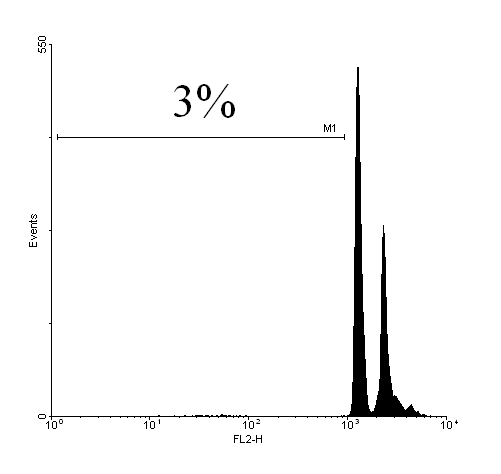 |
| NVP-AUY922 | 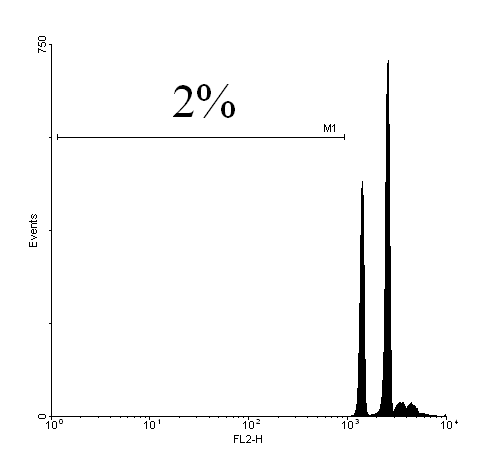 | 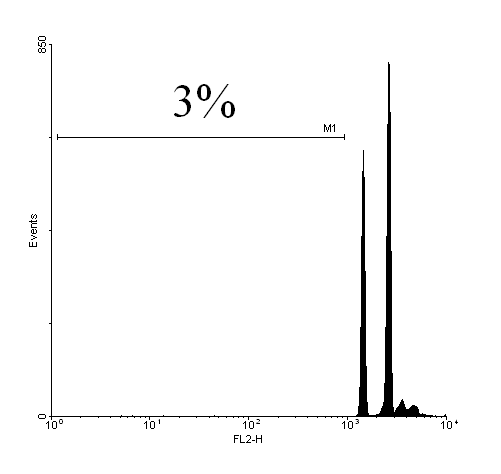 | 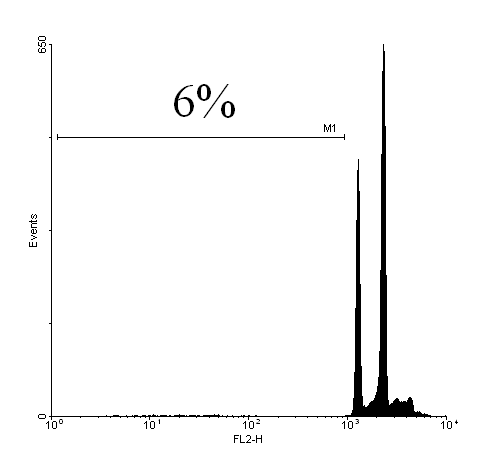 | 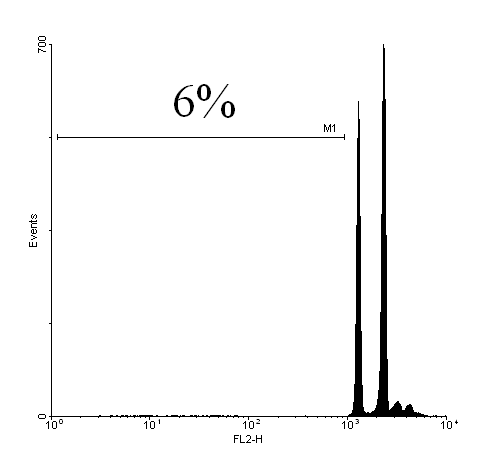 |
| NVP-BEP800 | 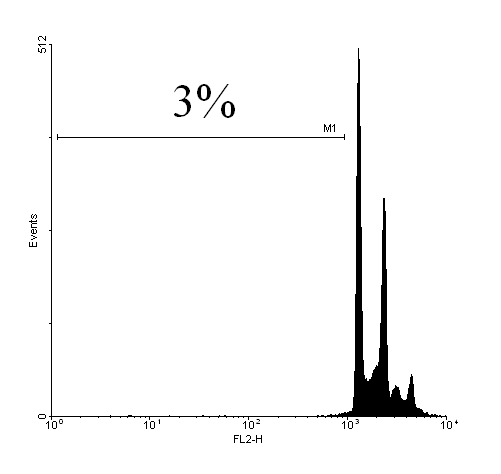 | 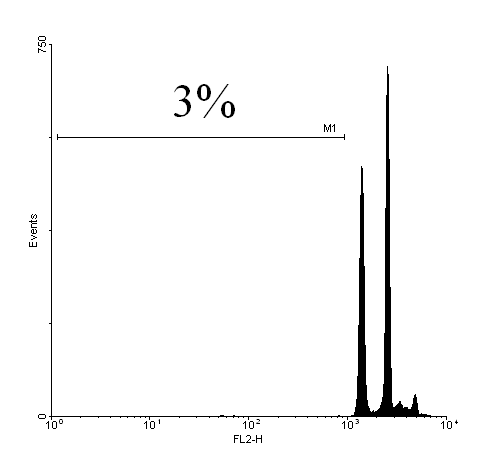 | 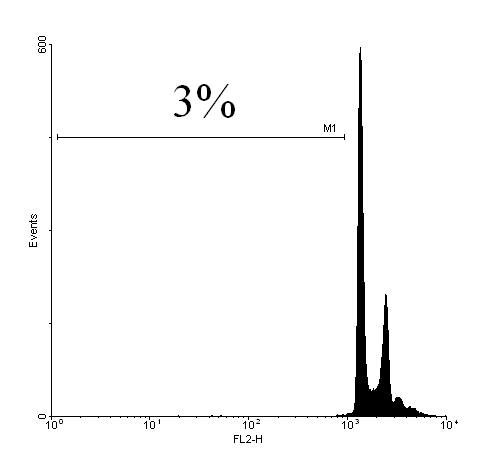 | 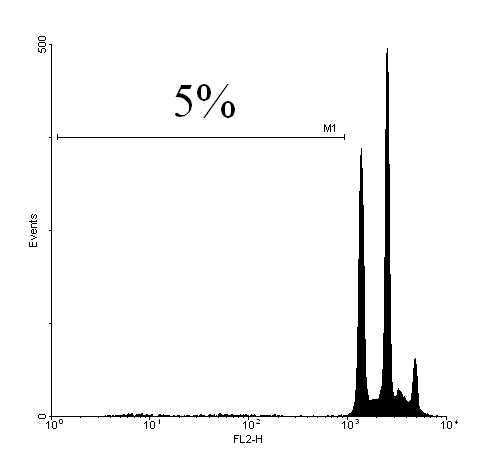 |
| 17-DMAG | 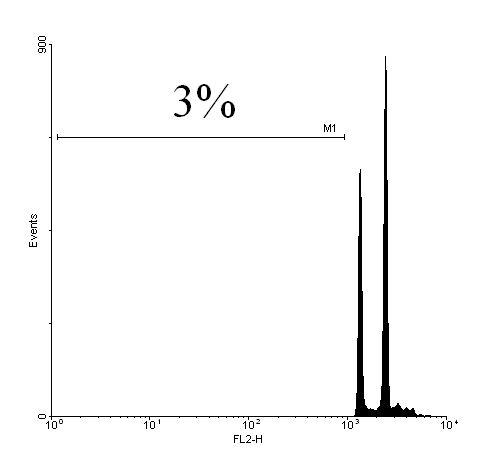 | 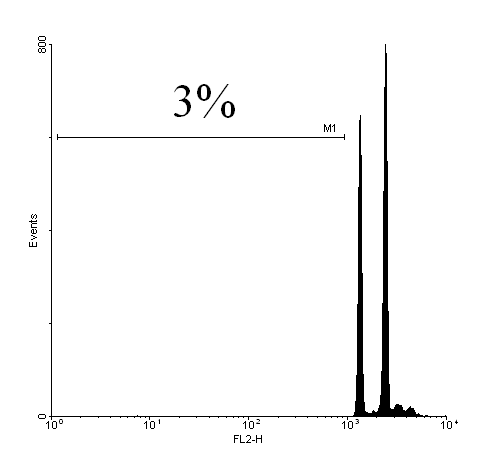 | 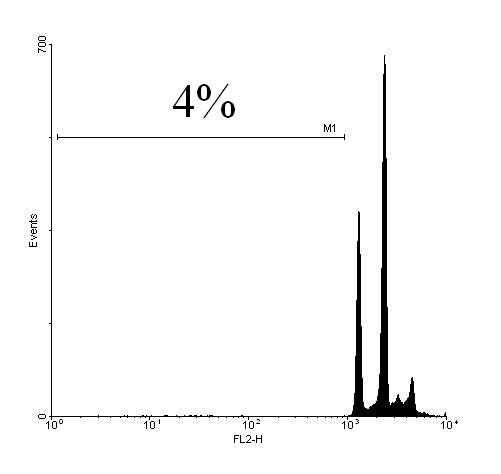 | 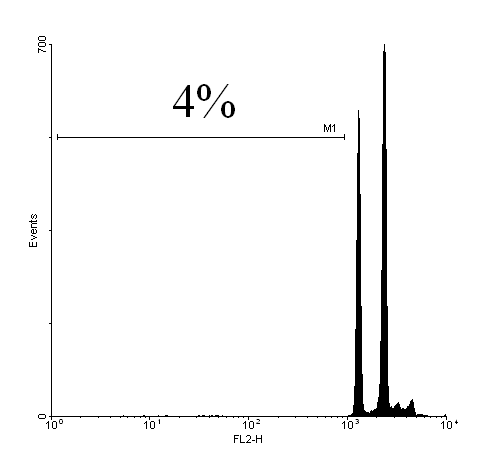 |

**Figure S2A.** Percentage of cells with hypodiploid DNA content and cellular debris in drug-treated and irradiated A549 cells measured flow cytometrically. Drug treated and irradiated (8 Gy) cells were cultivated for 24 and 48 h. At indicated time intervals the cells were detached with trypsin, treated with saponin and RNAse and analyzed for red fluorescence by flow cytometry after staining with PI. The samples include both floating and trypsinized cells. PI/DNA fluorescence was acquired in the logarithmic mode. The numbers denote the percentages of events for hypodiploid nuclei and debris in non-irradiated and irradiated cell samples, computed by means of the WinMDI Software.

Stingl et al. Supplementary Information

|  | 24 h | | 48 h | |
| --- | --- | --- | --- | --- |
|  | **0 Gy** | **8 Gy** | **0 Gy** | **8 Gy** |
| DMSO | 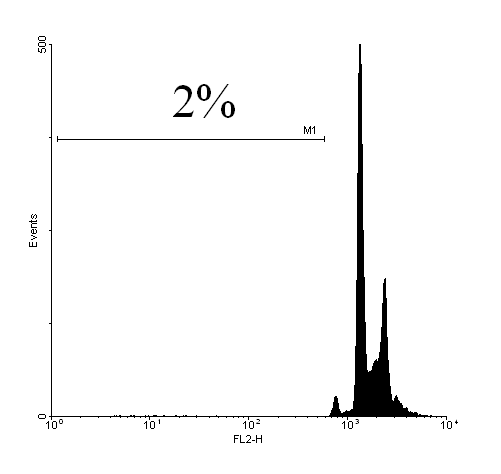 | 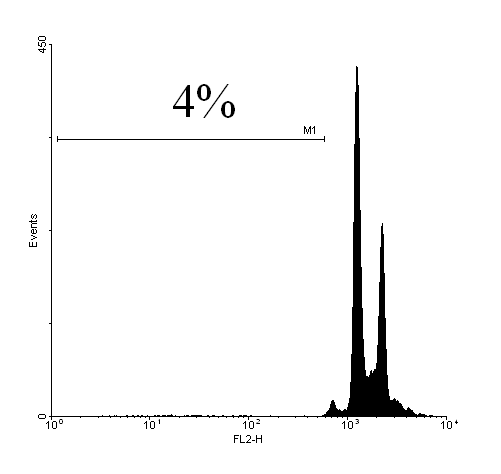 | 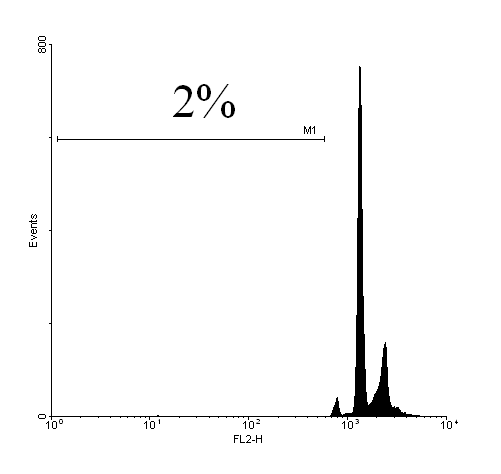 | 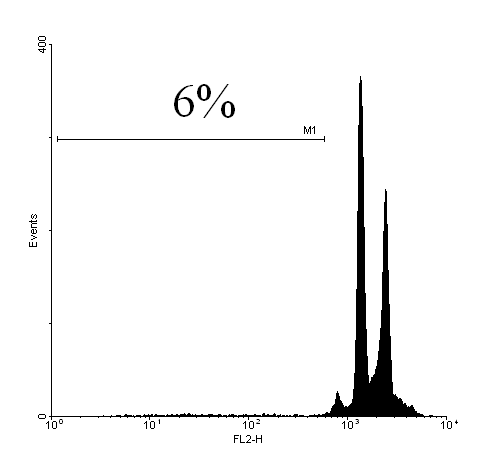 |
| NVP-AUY 922 | 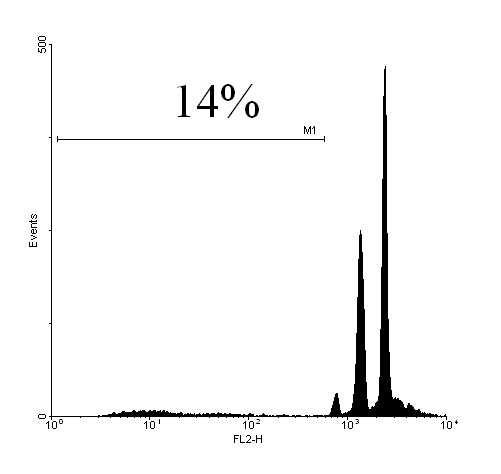 | 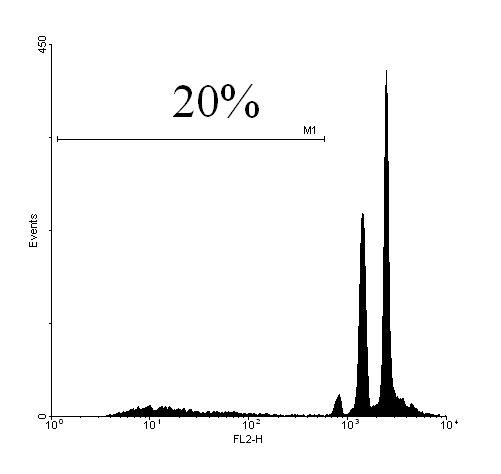 | 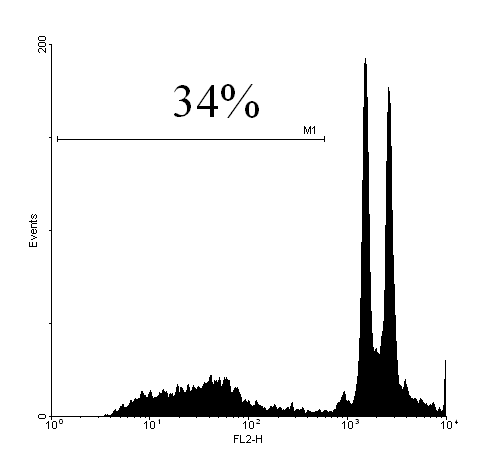 | 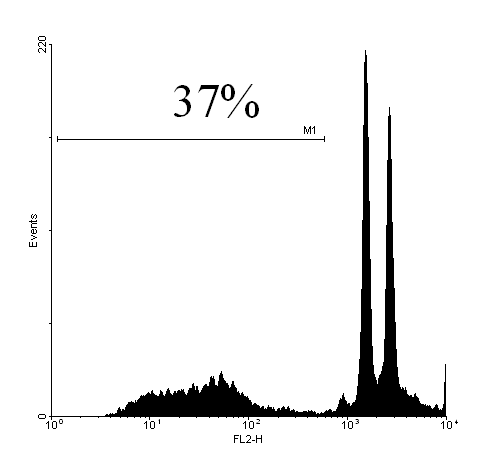 |
| NVP-BEP800 | 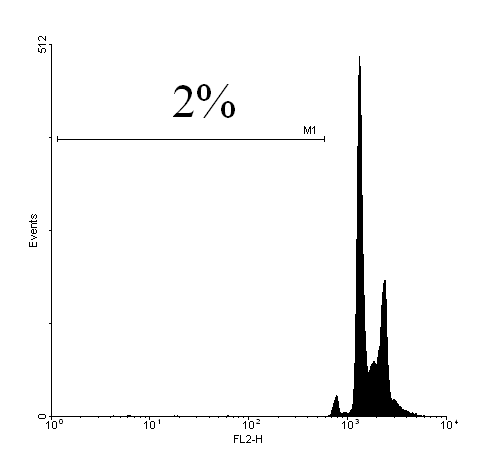 | 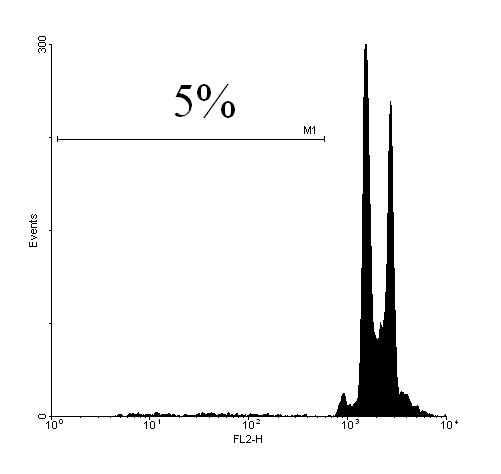 | 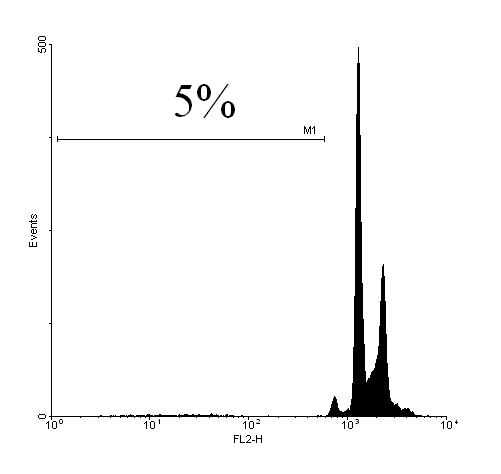 | 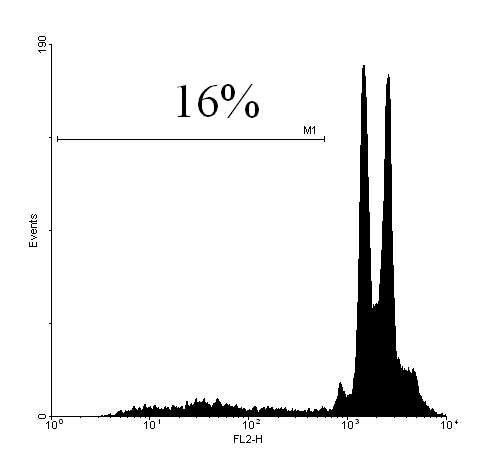 |
| 17-DMAG | 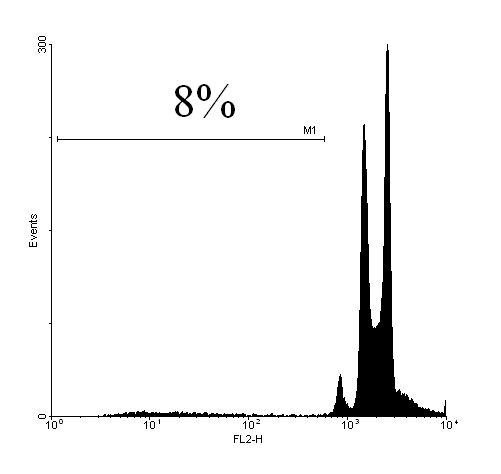 | 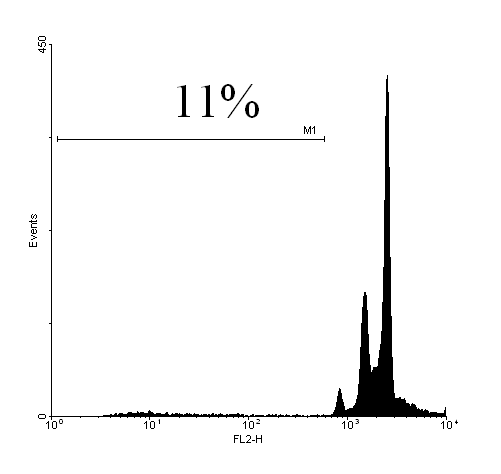 | 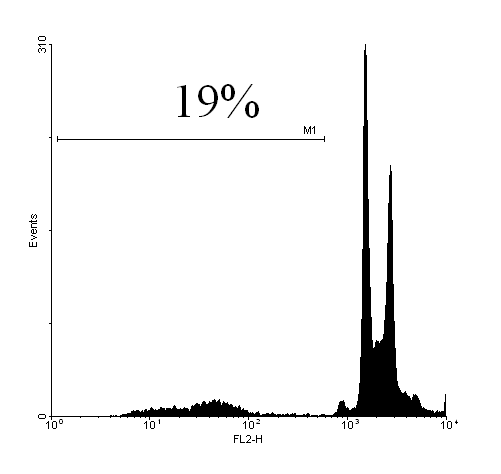 | 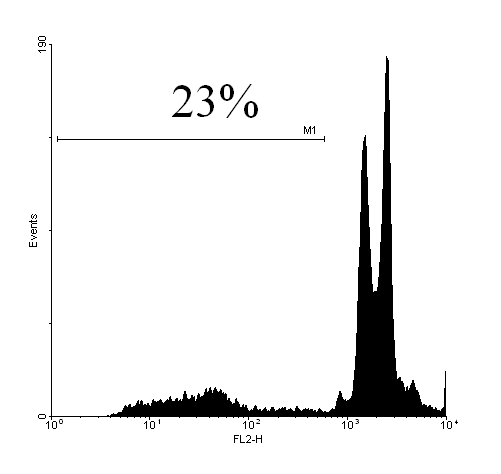 |

**Figure S2B.** Percentage of cells with hypodiploid DNA content and cellular debris in drug-treated and irradiated GaMG cells measured flow cytometrically. For details, *see* Legend to Fig. S2A.

Stingl et al. Supplementary Information

|  | 24 h | | 48 h | |
| --- | --- | --- | --- | --- |
|  | **0 Gy** | **8 Gy** | **0 Gy** | **8 Gy** |
| DMSO | 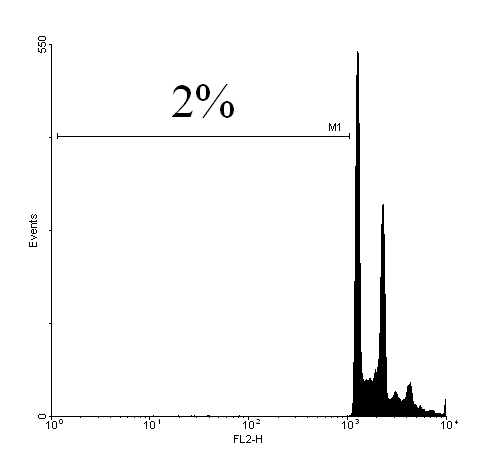 | 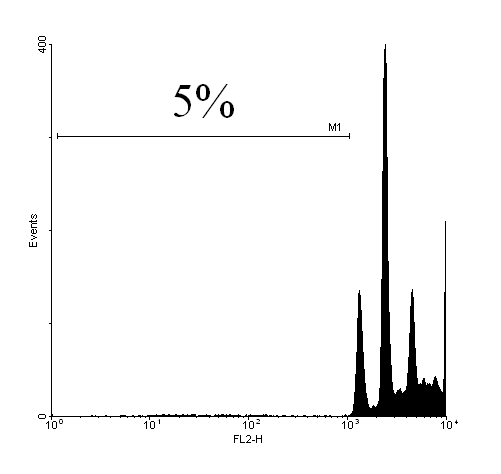 | 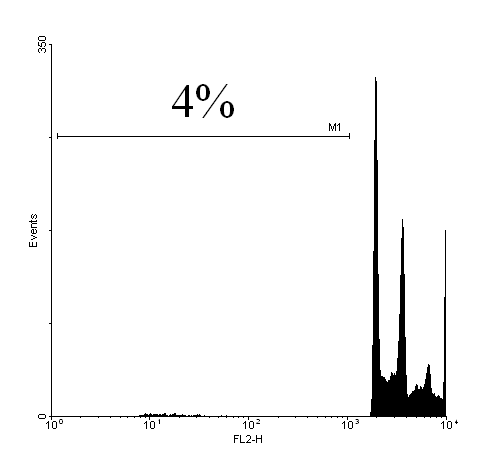 | 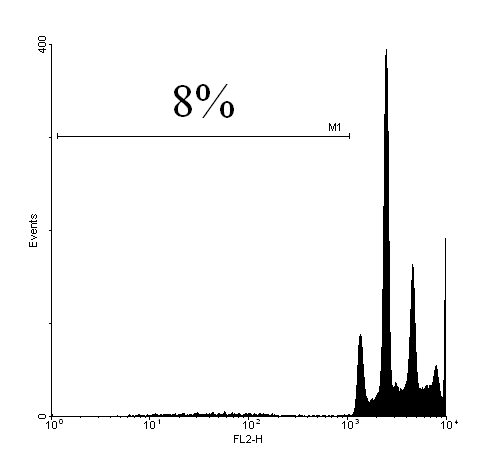 |
| NVP-AUY922 | 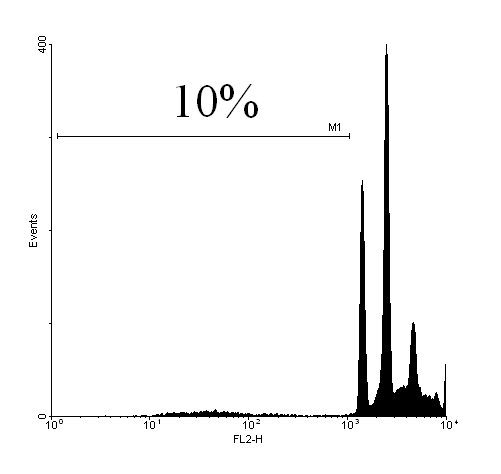 | 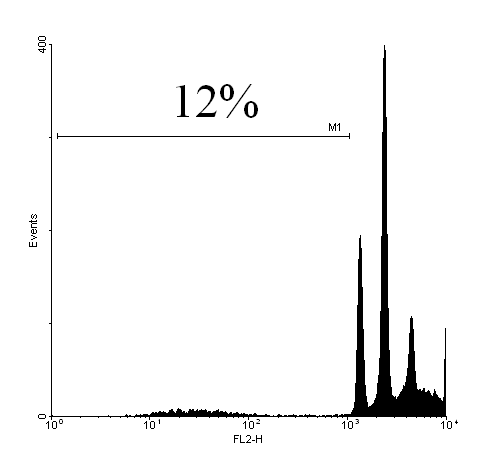 | 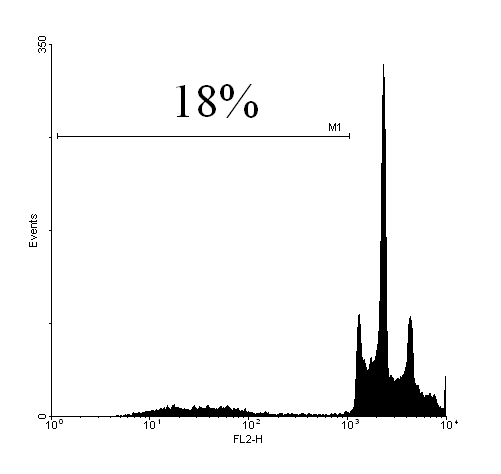 | 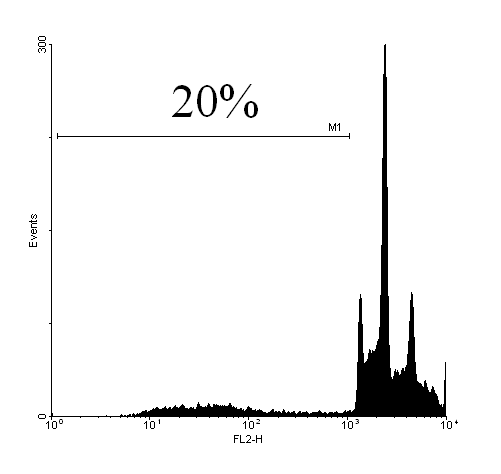 |
| NVP-BEP800 | 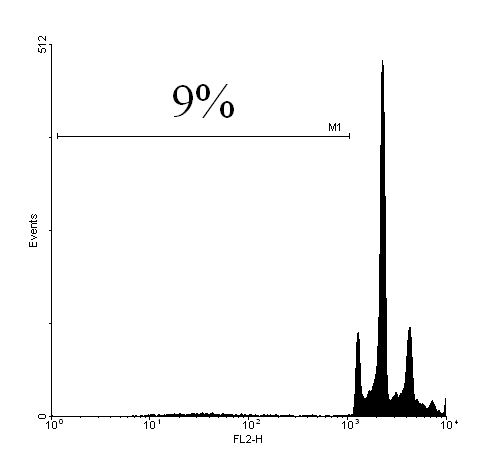 | 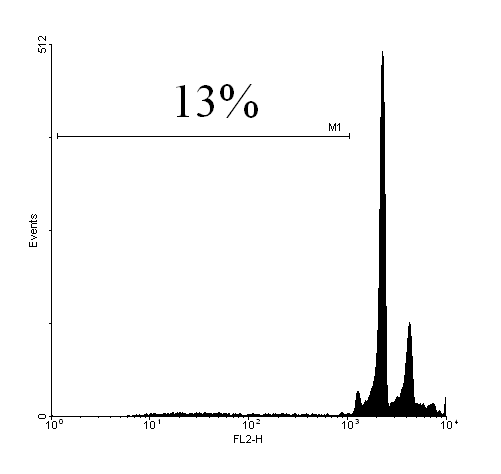 | 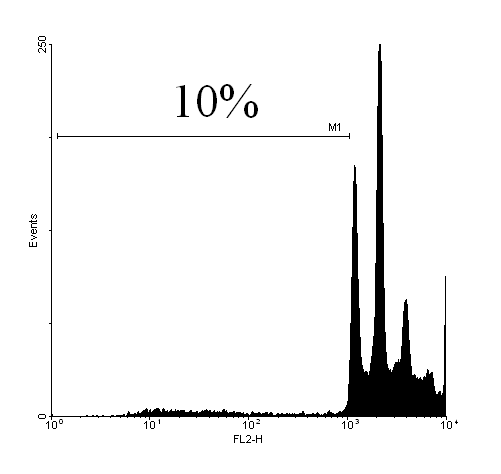 | 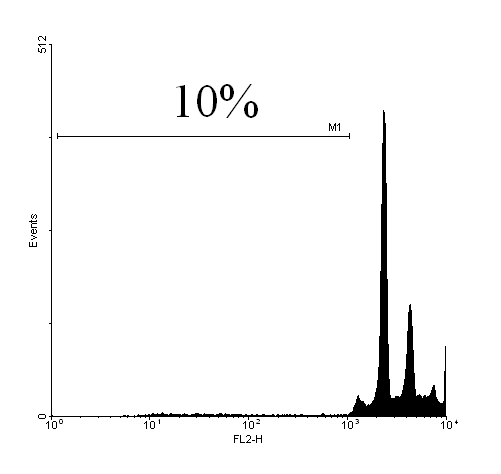 |
| 17-DMAG | 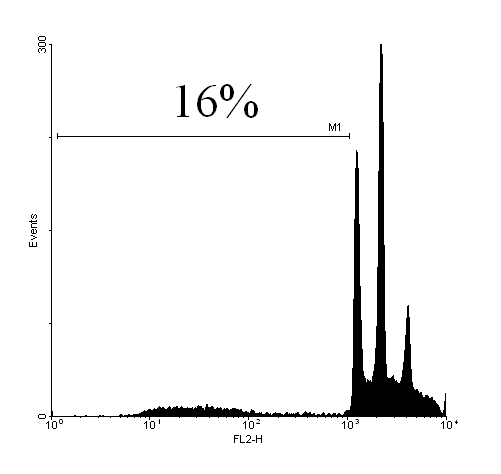 | 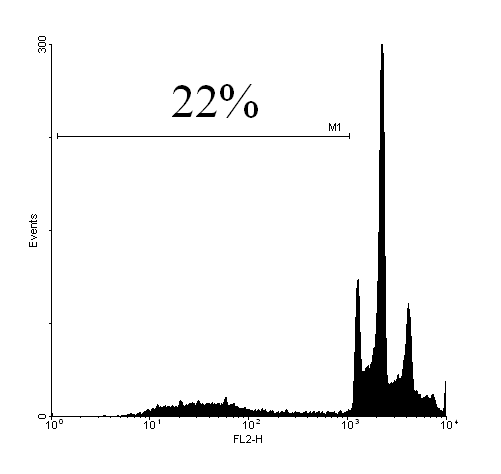 | 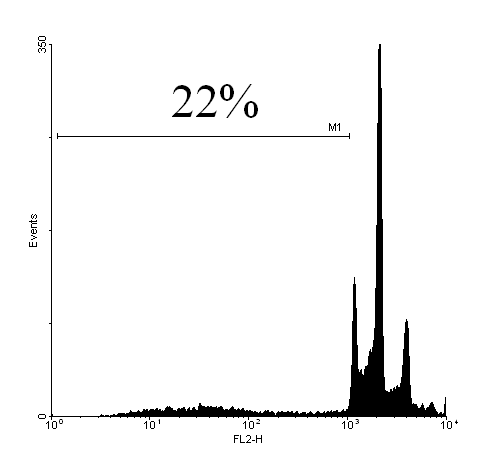 | 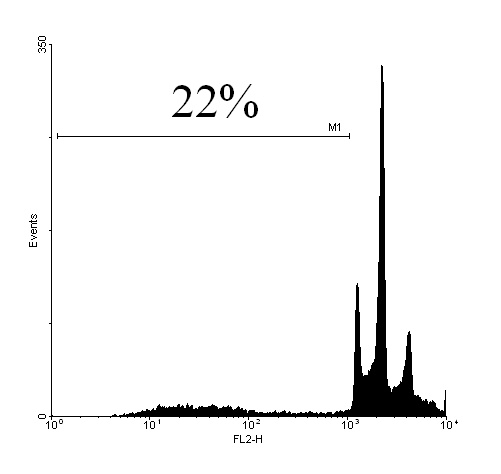 |

**Figure S2C.** Percentage of cells with hypodiploid DNA content and cellular debris in drug-treated and irradiated 4 tumor cell lines HT 1080 cells measured flow cytometrically. For details, *see* Legend to Fig. S2A.

Stingl et al. Supplementary Information

|  | 24 h | | 48 h | |
| --- | --- | --- | --- | --- |
|  | **0 Gy** | **8 Gy** | **0 Gy** | **8 Gy** |
| DMSO | 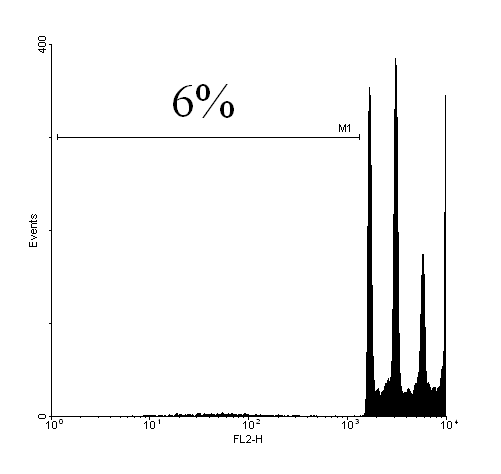 | 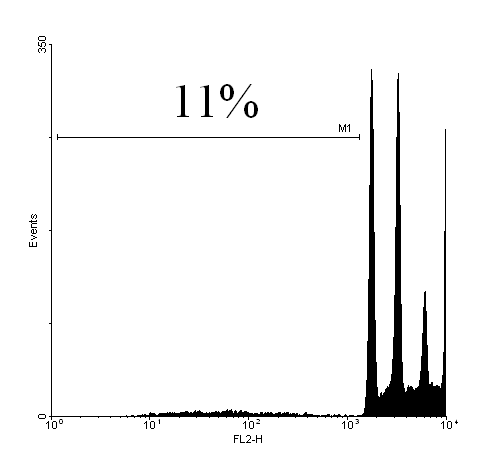 | 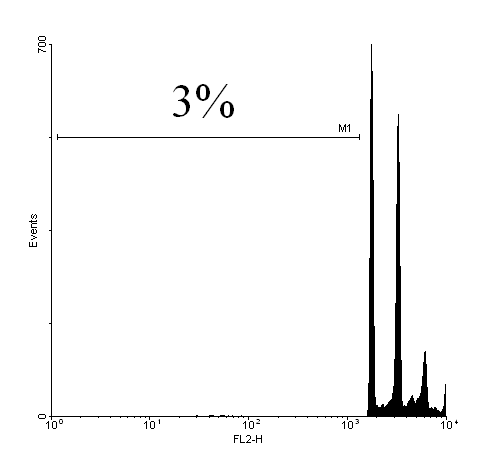 | 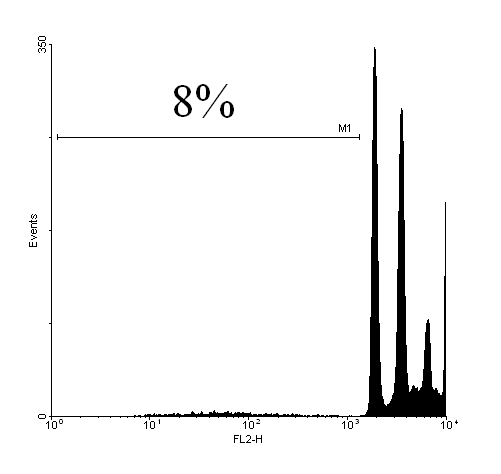 |
| NVP-AUY922 | 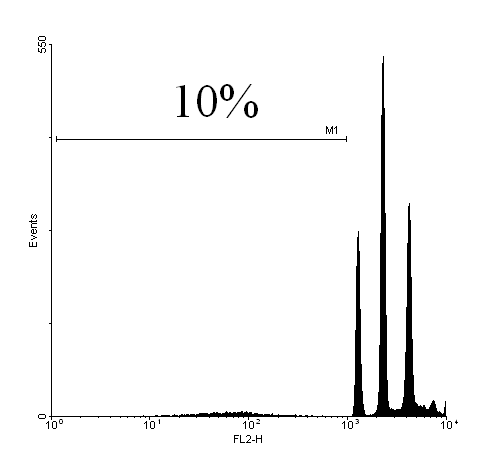 | 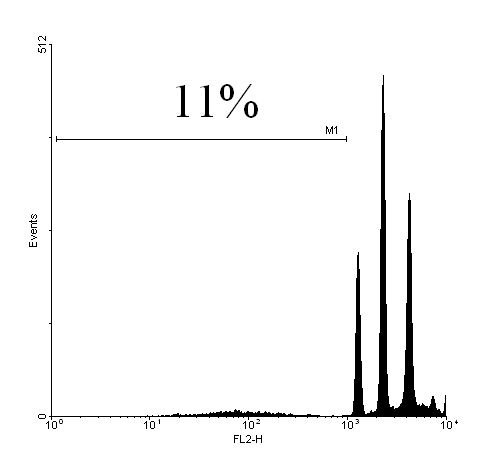 | 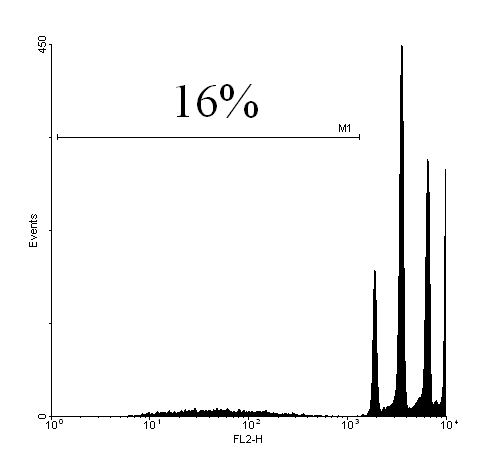 | 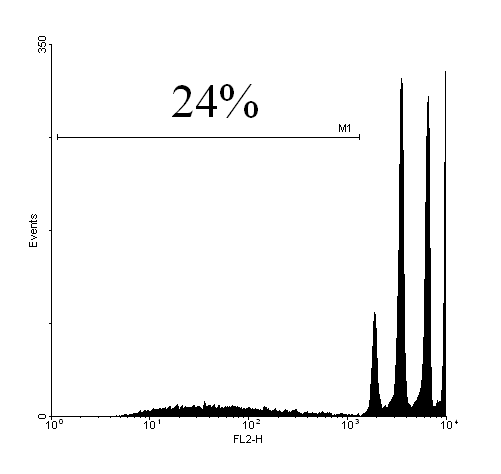 |
| NVP-BEP800 | 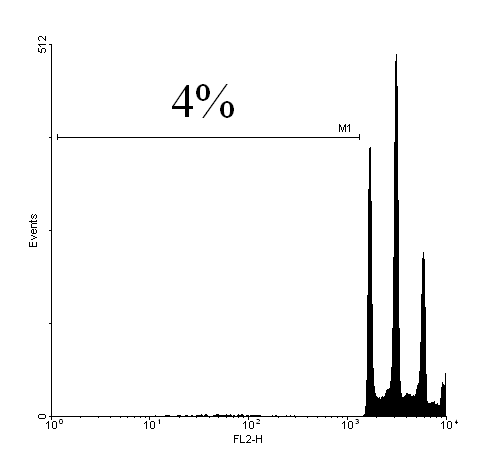 | 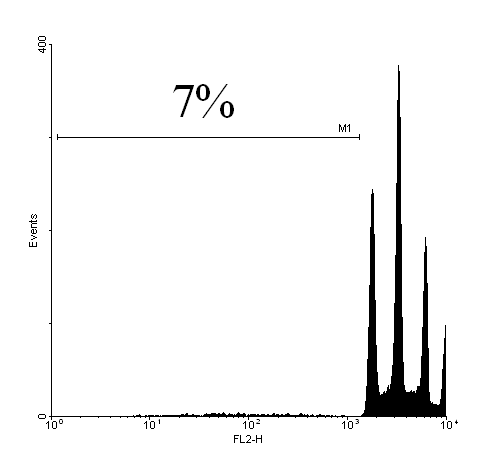 | 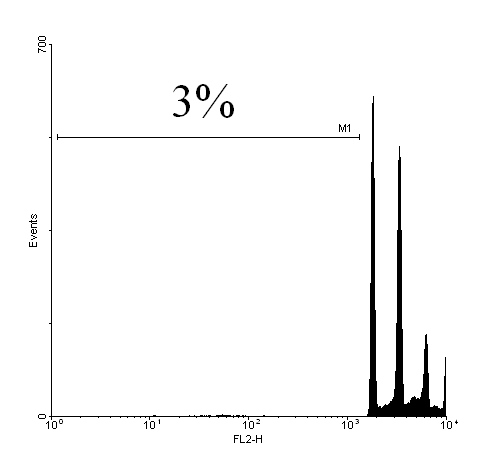 | 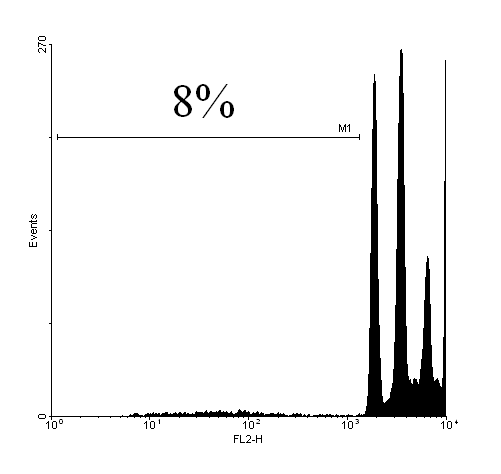 |
| 17-DMAG | 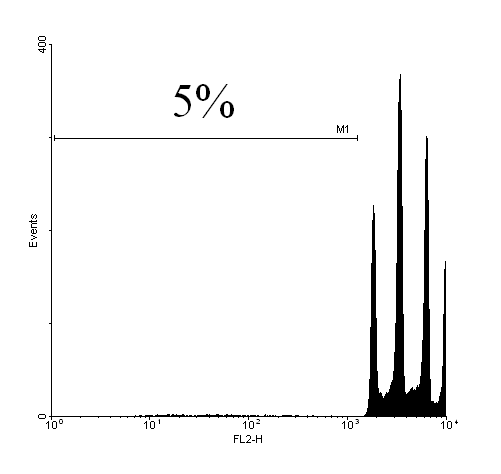 | 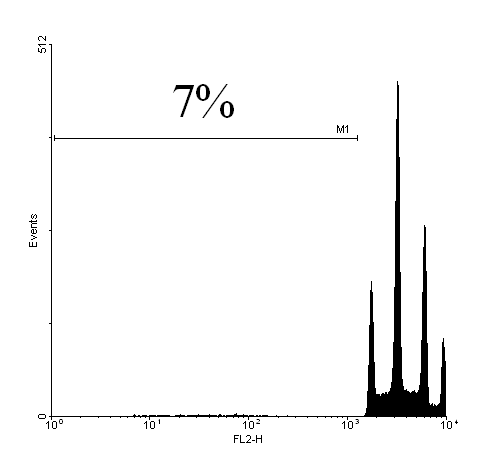 |  |  |

**Figure S2D.** Percentage of cells with hypodiploid DNA content and cellular debris in drug-treated and irradiated 4 tumor cell lines SNB19 cells measured flow cytometrically. For details, *see* Legend to Fig. S2A.

Stingl et al. Supplementary Information

**Figure** **S3**. Time courses of the expression of nuclear histone γH2AX in 4 drug-treated tumor cell lines, detected 30 min, 24 and 48 h post-irradiation with 8 Gy. The values (mean ± SD) are averaged over 3 independent experiments. To facilitate visual comparison, the modal γH2AX value in DMSO treated and irradiated sample is set to unity.

Stingl et al. Supplementary Information

**Figure S4A.** Effects of the Hsp90 inhibitors, IR and combined drug-IR treatment on the cell cycle-phase distribution in GaMG cells. Cells were drug treated and/or irradiated with 8 Gy, cultured for 24 and 48 h, fixed, permeabilized, stained with PI and analyzed for DNA content by flow cytometry using linear signal amplification. Deconvolution of DNA histograms was performed with ModFit Software. The numbers denote the percentage of cells in G1-, S- and G2/M phases and G2/G1 ratios in each cell sample. Arrows show the fraction of hyperdiploid cells. Filled and unfilled histograms represent irradiated and non-irradiated cells, respectively.

Stingl et al. Supplementary Information

**Figure S4B.** Effects of the Hsp90 inhibitors, IR and combined drug-IR treatment on the cell cycle-phase distribution in HT 1080 cells. For details, *see* Legend to Fig. S4A.

Stingl et al. Supplementary Information

**Figure S4C.** Effects of the Hsp90 inhibitors, IR and combined drug-IR treatment on the cell cycle-phase distribution in SNB19 cells. For details, *see* Legend to Fig. S4A.

Stingl et al. Supplementary Information

| **0 Gy** | | | | | | | **8 Gy** | | | | |  |
| --- | --- | --- | --- | --- | --- | --- | --- | --- | --- | --- | --- | --- |
| **DMSO** | **AUY922** | | **BEP800** | | **17DMAG** | | **DMSO** | **AUY922** | **BEP800** | **17DMAG** | |  |
|  | | | | | | |  | | | | | Cdk1 (34 kDa) |
| 0.8 | 0.7 | | 0.8 | | 0.7 | | 0.8 | 0.8 | 0.8 | 0.7 | | Cdk1/actin |
| 0.7 | 0 | | 0.2 | | 0 | | 0.9 | 0 | 0.2 | 0 | |  |
|  | | | | | | |  | | | | | Cdk2 (33 kDa) |
| 0.7 | 0.7 | | 0.7 | | 0.7 | | 0.8 | 0.7 | 0.7 | | 0.7 | Cdk2/actin |
|  | | | | | | |  | | | | | Cdk4 (30 kDa) |
| 1.0 | 0.8 | | 0.9 | | 0.7 | | 1.0 | 0.8 | 1.0 | 0.8 | | Cdk4/actin |
| 0.7 | 0.7 | | 0.7 | | 0.7 | | 0.7 | 0.7 | 0.8 | 0.8 | |  |
|  | | | | | | |  | | | | | pRb Ser780 (110 kDa) |
| 0.7 | | 0 | | 0.3 | | 0 | 0.6 | 0 | 0.4 | 0 | | pRb Ser780/actin |

**Figure S5A.** Effects of Hsp90 inhibitors on the expression of cell-cycle regulatory proteins in A549 tumor cell line. Total cell extracts were prepared 30 min after irradiation with 8 Gy, resolved by SDS-PAGE, blotted and immunostained according standard procedure.

Stingl et al. Supplementary Information

| **0 Gy** | | | | | | | **8 Gy** | | | | |  |
| --- | --- | --- | --- | --- | --- | --- | --- | --- | --- | --- | --- | --- |
| **DMSO** | **AUY922** | | **BEP800** | | **17DMAG** | | **DMSO** | **AUY922** | **BEP800** | **17DMAG** | |  |
|  | | | | | | |  | | | | | Cdk1 (34 kDa) |
| 1.1 | 0 | | 0.1 | | 0 | | 1.4 | 0 | 0.1 | 0 | | Cdk1/actin |
|  | | | | | | |  | | | | | Cdk2 (33 kDa) |
| 0.9 | 1.0 | | 0.9 | | 0.9 | | 1.1 | 1.1 | 0.9 | | 1.1 | Cdk2/actin |
|  | | | | | | |  | | | | | Cdk4 (30 kDa) |
| 1.1 | 0.8 | | 0.8 | | 0.8 | | 1.4 | 0.7 | 0.8 | 0.7 | | Cdk4/actin |
| 0.2 | 0.2 | | 0.3 | | 0.4 | | 0.4 | 0.3 | 0.3 | 0.2 | |  |
|  | | | | | | |  | | | | | pRb Ser780 (110 kDa) |
| 2.7 | | 0 | | 0.4 | | 0 | 2.7 | 0 | 0.5 | 0 | | pRb Ser780/actin |

**Figure S5B.** Effects of Hsp90 inhibitors on the expression of cell-cycle regulatory proteins in HT 1080 tumor cell line. Total cell extracts were prepared 30 min after irradiation with 8 Gy, resolved by SDS-PAGE, blotted and immunostained according standard procedure.

Stingl et al. Supplementary Information

| **0 Gy** | | | | | | | **8 Gy** | | | | |  |
| --- | --- | --- | --- | --- | --- | --- | --- | --- | --- | --- | --- | --- |
| **DMSO** | **AUY922** | | **BEP800** | | **17DMAG** | | **DMSO** | **AUY922** | **BEP800** | **17DMAG** | |  |
|  | | | | | | |  | | | | | Cdk1 (34 kDa) |
| 0.8 | 0.3 | | 0.2 | | 0.3 | | 0.8 | 0.3 | 0.2 | 0.2 | | Cdk1/actin |
|  | | | | | | |  | | | | | Cdk2 (33 kDa) |
| 0.6 | 0.7 | | 0.6 | | 0.7 | | 0.7 | 0.7 | 0.6 | | 0.6 | Cdk2/actin |
|  | | | | | | |  | | | | | Cdk4 (30 kDa) |
| 0.6 | 0.4 | | 0.5 | | 0.4 | | 0.6 | 0.4 | 0.4 | 0.3 | | Cdk4/actin |
| 0.5 | 0.5 | | 0.5 | | 0.5 | | 0.5 | 0.5 | 0.4 | 0.4 | |  |
|  | | | | | | |  | | | | | pRb Ser780 (110 kDa) |
|  | |  | | 0.2 | |  |  |  | 0.3 |  | | ppRb Ser780/actin |
| 0.7 | | 0.7 | | 0.7 | | 0.7 | 0.8 | 0.8 | 0.8 | 0.6 | | pRb Ser780/actin |

**Figure S5C.** Effects of Hsp90 inhibitors on the expression of cell-cycle regulatory proteins in SNB19 tumor cell line. Total cell extracts were prepared 30 min after irradiation with 8 Gy, resolved by SDS-PAGE, blotted and immunostained according standard procedure.

Stingl et al. Supplementary Information: Table S1

| **Cell line** | **Treatment** | **24 h** | | **48 h** | |
| --- | --- | --- | --- | --- | --- |
|  |  | **0 Gy** | **8 Gy** | **0 Gy** | **8 Gy** |
|  |  |  |  |  |  |
| **A549** | DMSO | 1.0±0.0 | 1.0±0.0 | 1.0±0.0 | 2.0±0.6 |
|  |  |  |  |  |  |
|  | NVP-AUY922 | 2.3±0.3 | 3.0±0.6 | 4.7±0.9 | 4.7±0.9 |
|  |  |  |  |  |  |
|  | NVP-BEP800 | 2.3±0.3 | 2.3±0.3 | 2.7±0.3 | 4.0±1.5 |
|  |  |  |  |  |  |
|  | 17-DMAG | 3.0±1.0 | 3.0±1.0 | 4.0±4.3 | 4.3±1.4 |
|  |  |  |  |  |  |
| **GaMG** | DMSO | 2.3±0.3 | 3.0±0.6 | 2.7±0.3 | 4.7±0.7 |
|  |  |  |  |  |  |
|  | NVP-AUY922 | 21±1.0 | 18±2.2 | 29±2.6 | 34±2.1 |
|  |  |  |  |  |  |
|  | NVP-BEP800 | 3.0±0.6 | 5.6±0.7 | 6.7±1.2 | 16.7±4 |
|  |  |  |  |  |  |
|  | 17-DMAG | 12±3.0 | 14±3.7 | 22±1.7 | 26±2.6 |
|  |  |  |  |  |  |
| **HT1080** | DMSO | 3.0±0.6 | 6.0±1.1 | 3.7±0.7 | 8.3±0.9 |
|  |  |  |  |  |  |
|  | NVP-AUY922 | 10±1.1 | 11±2.0 | 13±1.4 | 17±3.5 |
|  |  |  |  |  |  |
|  | NVP-BEP800 | 5.3±0.9 | 7.3±0.3 | 7.7±1.2 | 13±1.8 |
|  |  |  |  |  |  |
|  | 17-DMAG | 6.0±1.0 | 8.3±0.9 | 8.3±0.9 | 15±0.3 |
|  |  |  |  |  |  |
| **SNB19** | DMSO | 1.7±0.3 | 4.0±0.6 | 3.7±0.3 | 7.0±0.6 |
|  |  |  |  |  |  |
|  | NVP-AUY922 | 10±0.3 | 12±0.3 | 17±1.0 | 17±1.8 |
|  |  |  |  |  |  |
|  | NVP-BEP800 | 11±0.9 | 13±1.1 | 11±0.7 | 15±2.6 |
|  |  |  |  |  |  |
|  | 17-DMAG | 12±1.8 | 16±4.2 | 19±2.2 | 23±0.7 |

**Table S1**. Percentage of cells with hypodiploid DNA content and cellular debris after pretreatment (200 nM, 24 h) with different Hsp90 inhibitors followed by irradiation (8 Gy) and subsequent incubation for 24 and 48 h

Stingl et al. Supplementary Information: Table S2

| **Cell line** | **G0/G1, %** | **S, %** | **G2/M, %** | **G2/G1** |
| --- | --- | --- | --- | --- |
| **A549** |  |  |  |  |
| DMSO**^b^** | 53±3**^a^** | 36±2 | 11±4 | 0.2 |
| NVP-AUY922 | 53±5 | 4±1 | 43±4 | 0.8 |
| NVP-BEP800 | 39±12 | 11±5 | 51±8 | 1.3 |
| 17-DMAG | 53±7 | 3±1 | 43±6 | 0.8 |
| **GaMG** |  |  |  |  |
| DMSO | 52±3 | 34±6 | 14±3 | 0.3 |
| NVP-AUY922 | 38±6 | 9±7 | 53±8 | 1.4 |
| NVP-BEP800 | 36±4 | 17±6 | 47±6 | 1.3 |
| 17-DMAG | 39±8 | 14±3 | 47±9 | 1.2 |
| **HT1080** |  |  |  |  |
| DMSO | 43±1 | 22±1 | 35±2 | 0.8 |
| NVP-AUY922 | 42±6 | 4±2 | 54±6 | 1.3 |
| NVP-BEP800 | 44±3 | 7±1 | 49±3 | 1.1 |
| 17-DMAG | 41±8 | 5±2 | 53±8 | 1.3 |
| **SNB19** |  |  |  |  |
| DMSO | 41±6 | 36±3 | 22±3 | 0.5 |
| NVP-AUY922 | 32±0 | 11±1 | 57±1 | 1.8 |
| NVP-BEP800 | 34±4 | 17±3 | 50±3 | 1.5 |
| 17-DMAG | 33±1 | 10±2 | 57±1 | 1.7 |

**Table S2.** Cell cycle-phase distribution in 4 tumor cell lines pretreated with different Hsp90 inhibitors (200 nM, 24 h)

Stingl et al. Supplementary Information: Table S3

| **Cell line** | **Dose (Gy)** | **24 h post-irradiation** | | | | **48 h post-irradiation** | | | |
| --- | --- | --- | --- | --- | --- | --- | --- | --- | --- |
|  |  | **G0/G1**  **(%)** | **S**  **(%)** | **G2/M**  **(%)** | **G2/G1** | **G0/G1**  **(%)** | **S**  **(%)** | **G2/M**  **(%)** | **G2/G1** |
| **A549-**DMSO | 0 | 59±3 | 30±2 | 11±1 | 0.2 | 82±4 | 10±3 | 8±2 | 0.1 |
|  | 8 | 64±3 | 8±1 | 29±2 | 0.5 | 68±2 | 5±4 | 27±4 | 0.4 |
| NVP-AUY922 | 0 | 42±5 | 3±1 | 54±5 | 1.3 | 35±4 | 15±5 | 50±4 | 1.4 |
|  | 8 | 43±5 | 3±1 | 55±6 | 1.3 | 44±2 | 6±1 | 50±2 | 1.1 |
| NVP-BEP800 | 0 | 49±3 | 32±2 | 18±3 | 0.4 | 68±6 | 21±4 | 11±3 | 0.2 |
|  | 8 | 46±5 | 6±1 | 48±5 | 1.0 | 46±3 | 11±2 | 44±3 | 1.0 |
| 17-DMAG | 0 | 39±4 | 11±2 | 50±4 | 1.3 | 33±1 | 14±3 | 53±2 | 1.6 |
|  | 8 | 43±3 | 5±1 | 52±3 | 1.2 | 42±2 | 8±1 | 50±3 | 1.2 |
| **GaMG-**DMSO | 0 | 52±3 | 31±1 | 17±3 | 0.3 | 62±3 | 23±4 | 16±3 | 0.3 |
|  | 8 | 49±5 | 23±5 | 28±1 | 0.6 | 42±6 | 29±8 | 29±5 | 0.7 |
| NVP-AUY922 | 0 | 48±13 | 3±1 | 50±13 | 1.0 | 47±7 | 27±5 | 26±4 | 0.6 |
|  | 8 | 48±12 | 3±1 | 49±12 | 1.0 | 40±9 | 21±8 | 39±11 | 1.0 |
| NVP-BEP800 | 0 | 38±3 | 34±5 | 28±3 | 0.7 | 56±7 | 28±5 | 16±3 | 0.3 |
|  | 8 | 28±5 | 25±6 | 47±4 | 1.7 | 38±7 | 26±5 | 36±5 | 1.0 |
| 17-DMAG | 0 | 33±4 | 35±4 | 32±8 | 1.0 | 40±4 | 35±2 | 25±5 | 0.6 |
|  | 8 | 25±5 | 38±2 | 37±5 | 1.5 | 29±4 | 31±5 | 40±4 | 1.4 |
| **HT1080-**DMSO | 0 | 39±9 | 24±9 | 37±15 | 0.9 | 41±8 | 11±3 | 48±10 | 1.2 |
|  | 8 | 49±16 | 12±5 | 39±11 | 0.8 | 48±8 | 8±1 | 48±8 | 1.0 |
| NVP-AUY922 | 0 | 35±5 | 11±15 | 54±11 | 1.5 | 32±8 | 12±1 | 56±9 | 1.8 |
|  | 8 | 41±9 | 6±5 | 53±14 | 1.3 | 32±11 | 7±2 | 61±10 | 1.9 |
| NVP-BEP800 | 0 | 31±11 | 23±3 | 46±13 | 1.5 | 33±8 | 13±4 | 54±12 | 1.6 |
|  | 8 | 38±11 | 14±2 | 48±10 | 1.3 | 33±7 | 7±3 | 60±9 | 1.8 |
| 17-DMAG | 0 | 41±6 | 11±8 | 47±13 | 1.1 | 26±2 | 15±3 | 59±4 | 2.3 |
|  | 8 | 45±11 | 5±3 | 49±14 | 1.1 | 31±7 | 9±4 | 59±5 | 1.9 |
| **SNB19-**DMSO | 0 | 43±7 | 33±7 | 24±1 | 0.6 | 45±4 | 30±9 | 26±6 | 0.6 |
|  | 8 | 35±11 | 7±4 | 58±13 | 1.7 | 22±5 | 18±5 | 60±8 | 2.7 |
| NVP-AUY922 | 0 | 32±1 | 8±1 | 59±2 | 1.8 | 16±1 | 30±4 | 54±5 | 3.4 |
|  | 8 | 33±2 | 9±2 | 57±1 | 1.7 | 11±3 | 28±12 | 60±15 | 5.5 |
| NVP-BEP800 | 0 | 12±3 | 24±5 | 64±8 | 5.3 | 21±5 | 24±6 | 55±11 | 2.6 |
|  | 8 | 3±2 | 21±4 | 76±5 | 25 | 3±3 | 14±6 | 82±9 | 27 |
| 17-DMAG | 0 | 32±1 | 17±4 | 51±5 | 1.6 | 10±5 | 29±11 | 60±11 | 6.0 |
|  | 8 | 31±2 | 17±4 | 52±6 | 1.7 | 10±4 | 28±9 | 62±8 | 6.2 |

**Table S3**. Cell cycle-phase distribution in 4 tumor cell lines pretreated with different Hsp90 inhibitors detected 24 h and 48 h post-irradiation (8 Gy)
